# Establishment of a promoter-based chromatin architecture on recently replicated DNA can accommodate variable inter-nucleosome spacing

**DOI:** 10.1093/nar/gkw331

**Published:** 2016-04-22

**Authors:** Ross T. Fennessy, Tom Owen-Hughes

**Affiliations:** Centre for Gene Regulation and Expression, School of Life Sceinces, University of Dundee, Dundee, DD1 5EH, UK

## Abstract

Nucleosomes, the fundamental subunits of eukaryotic chromatin, are organized with respect to transcriptional start sites. A major challenge to the persistence of this organization is the disassembly of nucleosomes during DNA replication. Here, we use complimentary approaches to map the locations of nucleosomes on recently replicated DNA. We find that nucleosomes are substantially realigned with promoters during the minutes following DNA replication. As a result, the nucleosomal landscape is largely re-established before newly replicated chromosomes are partitioned into daughter cells and can serve as a platform for the re-establishment of gene expression programmes. When the supply of histones is disrupted through mutation of the chaperone Caf1, a promoter-based architecture is generated, but with increased inter-nucleosomal spacing. This indicates that the chromatin remodelling enzymes responsible for spacing nucleosomes are capable of organizing nucleosomes with a range of different linker DNA lengths.

## INTRODUCTION

The genomes of eukaryotes exist as chromatin. The fundamental subunit of chromatin, the nucleosome is not a static structure, but can be reconfigured dynamically. For example, variant histones can be incorporated into nucleosomes and the histone polypeptides themselves subject to extensive post-translational modification. In combination, such changes have led to the identification of distinct chromatin states (1–3). Chromatin states are often conserved through cell divisions, and recent studies have shown that different types of histone modification are restored at different rates (4,5). However, the processes that underlie this are poorly understood.

The positioning of nucleosomes is non-random and influences access to underlying regulatory DNA sequences (6,7). The separation of DNA strands during replication requires dissociation of histones and raises the question of how nucleosomes are reorganized to the positions that are optimal for their functions in gene regulation. Previous studies have indicated that following replication, chromatin exists in a state that is distinct to mature chromatin. For example pulse chase radiolabelling has been used to show that chromatin is more sensitive to nuclease digestion 1 min following replication, but matures within about 10 min (8–12). Rapid reassembly of nucleosomes is supported by electron micrographs showing nucleosomes assembled close to replication origins (13). Subsequently, analysis of the regions protected from psoralen cross-linking showed that nucleosomes are assembled within 250 bp of replication forks (14–16). As DNA replication proceeds at several kilobytes per minute (17), this indicates that nucleosomes are reassembled within seconds. A related approach was then used to show that nucleosomes at the rDNA locus are assembled at positions in nascent chromatin that are similar to those observed in mature chromatin 600 bp from a replication fork (18).

Since these studies were carried out further progress has been made towards understanding how nucleosomes are organized on a genome scale. In budding yeast it has been observed that nucleosomes are organized with respect to coding genes (19,20). In some locations the underlying structural properties of DNA may contribute to nucleosome organization. However, this effect is likely to be greatest at the nucleosome depleted regions within the vicinity of promoters (21). Trans acting factors are implicated in the establishment of the regularly spaced arrays of nucleosomes over coding regions. Amongst these, a subset of chromatin remodelling adenosine triphosphatase (ATPases) with the biochemical capability to generate regularly spaced arrays of nucleosomes are attractive candidates (22,23). Further support for this stems from the observation that deletion of combinations of ISWI and Chd1 enzymes results in the loss of nucleosome organization over coding regions (24–26).

Although it is clear that adenosine triphosphate (ATP)-dependent chromatin remodelling enzymes act to organize nucleosomes over coding regions, it is less clear when this occurs or how long it takes. The fact that nucleosomes are organized across coding regions suggests that nucleosomes organization is coupled to transcription. Supporting this the key enzymes ATPases associated with nucleosome organization are both linked to elongating RNA polymerase. Chd1 through its interaction with the RNA Polymerase II-associated factor (PAF) complex (27) and Isw1b through its interaction with the coding region histone modification H3 K36me3 (28). However, following inhibition of transcription promoter-based chromatin architecture persists for 20 min becoming perturbed but not lost after 120 min (29). This indicates that ongoing transcription is not required to maintain nucleosome organization. In addition, it has been observed that yeast extracts that do not support transcription are capable of partially restoring promoter based chromatin architecture (30). From these observations, it is not clear when nucleosome organization is established over the majority of coding regions, and especially how long it takes for this to occur following the disassembly of nucleosomes coupled to the transit of DNA polymerase.

If replication origins were used with high efficiency and identical timing in all cells within a population, it would be possible to study nascent chromatin by isolating chromatin from synchronized cultures. However, origin use and timing varies (31), possibly explaining why intermediates in chromatin reassembly are not detected in the bulk chromatin of synchronized cultures (32,33). To address this, we have developed approaches to specifically enrich for recently replicated DNA. Using these we show that the majority of nucleosomes are aligned to promoters within the minutes following replication. This supports the existence of a transcription independent pathway capable of organizing nucleosomes over gene bodies. This provides a means of re-establishing nucleosome organization on newly replicated chromosomes prior to their segregation into daughter cells. As a result genome scale nucleosome organization can be propagated through mitotic cell divisions.

## MATERIALS AND METHODS

### Stable isotope labelling

Differential mass labelling was performed by growth in heavy medium (34) containing D-glucose-^13^C_6_,1,2,3,4,5,6,6-d7 (Cambridge isotope laboratories) and Ammonium-^15^N sulphate (Sigma-Aldrich). Cells were grown in heavy media to an OD_660_ of 0.66 at 30°C. The α-factor mating pheromone was added to a final concentration of 50 ng/ml for 1 h 30 min. Cell morphology was checked by light microscopy to ensure cells were in M or G_1_ phase. Cells were collected and washed on cellulose filter membranes with 800 ml of warm YPAD. Cells were re-suspended in 350 ml of YPAD containing 50 ng/ml α-factor and grown for 60 min at 30°C. Cell morphology was again checked by light microscopy for shmoo formation representative of G_1_ arrest. Cells were filter washed with 800 ml of YPAD and released into 350 ml of YPAD (isotopically light) S-phase medium at 23°C. Approximately 50 ml of cells were collected at defined time points and treated with formaldehyde to allow fixation for subsequent chromatin digestion.

### CsCl gradient ultracentrifugation

A solution of CsCl (sigma) and T_10_E_100_ was made to a starting density of 1.4 g/g (CsCl/ T_10_E_100_). A total of 90 μl (in T_10_E_0.1_, pH 7.5) of MNase digested, differentially mass labelled DNA was mixed with 9.3564 g of CsCl solution and sealed in a 5.1 ml ultracentrifugation tube (Beckman Coulter). Centrifugation (Vti 65.2 rotor) was performed sequentially at 65 000 rpm for 50 h, 50 000 rpm for 18 h, 28 000 rpm for 3.5 h and brought to rest with the slow brake setting applied.

Ultracentrifugation tubes were fixed to a retort stand and pierced at the base and then top with a small bore needle. Mineral oil was pumped in the top of the ultracentrifugation tube forcing drop wise elution from the tube at a rate of ∼400 μl/min. A total of 250 μl of CsCl gradient was collected per fraction allowing collection of ∼20 fractions per gradient. Gradient fractions were subsequently dialysed against water (50 ml) on a floating dialysis membrane (Millipore) for 60 min. Fractions 9 and 17 were chosen to represent the non-replicated (HH) and replicated (HL) portions of the gradient respectively.

### EdU labelling in synchronized cultures

Cultures were grown to an OD_660_ of 0.66 at 30°C in YPAD and synchronized with α-factor. Cells were filter washed with YPAD and released into YPAD medium containing 50 μM EdU at 23°C. Cells were harvested at defined time points and were fixed with formaldehyde for subsequent MNase digestion.

### EdU labelling in asynchronous cultures

Cultures were grown to an OD_660_ of 0.8 at 23°C in YPAD. EdU was added to a final concentration of 100 μM EdU. Cells were harvested at defined time points and fixed with formaldehyde for subsequent MNase digestion.

### Biotinylation and isolation of EdU labelled nascent DNA

Biotin azide was attached to EdU labelled DNA using the Click-iT^®^ Nascent RNA Capture Kit (Invitrogen, C10365). EdU labelled DNA replaced EU labelled RNA in the protocol. Isolation of biotinylated DNA was achieved using Dynabeads^®^ MyOne™ Streptavidin T1 (Invitrogen).

### Chromatin digestion and deep sequencing

Cells were cross-linked by addition of formaldehyde to a final concentration of 1% v/v for 10 min at room temperature (RT). Crosslinking was quenched with addition of 2.5 M glycine to a final concentration of 0.125 M and cells were further incubated for another 5 min at RT. Crosslinked cells were washed 3× with ice cold Tris-buffered saline (20mM Tris pH 7.5, 120 mM NaCl). Cells were mechanically lysed according to (35) and digested using micrococcal nuclease (MNase) according to (36). MNase titrations were selected to obtain largely mononucleosomal DNA with larger nucleosomal DNA fragments apparent. Nucleosomal DNA was prepared to create a library for paired end deep sequencing on Illumina platforms. Briefly, DNA was blunt ended, A-tailed and ligated to Illumina genomic adapters, followed by a final polymerase chain reaction with a size-selecting gel purification. Sequencing data is deposited at ENA ref PRJEB13217 (to be released upon acceptance for publication). Supplementary Table S1 provides a summary of the datasets released. Reads were mapped to the genome using bowtie (37). Representation of reads across individual loci was performed using IGB (38). Data was then analysed using custom python scripts included as Supplementary Data. For average plots surrounding multiple reference points, each value was divided by the sum of reads for each dataset as a means of normalization as illustrated in the python script accompanying the supplemental materials. Where applied, data was smoothed using a 75 bp moving average. For plots of nucleosomal reads across whole chromosomes, data was twice smoothed using a 10 000 bp moving average.

### Imaging of EdU labelled nascent DNA

Cultures were grown to an OD_660_ of 0.5 at 23°C in YPAD. EdU was added to a concentration of 100 μM for defined time points. Cells were fixed with 2% formaldehyde for 30 min and wash 3× with phosphate buffered saline (PBS). Cells were incubated with 0.5% triton x-100 for 25 min. Cells were then washed 2× with 3% bovine serum albumin (BSA) in PBS. Cells were further processed for the Click-iT EdU reaction as described in the protocol C10337 (Invitrogen). Subsequently cells were washed 2× with 0.1% tween in PBS and 2× finally with 3% BSA in PBS. The images were acquired with widefield microscopy using the OMX Blaze platform.

## RESULTS

### Affinity purification of EdU containing nucleosomal DNA provides a means of studying chromatin within minutes of replication

**Figure 1.**
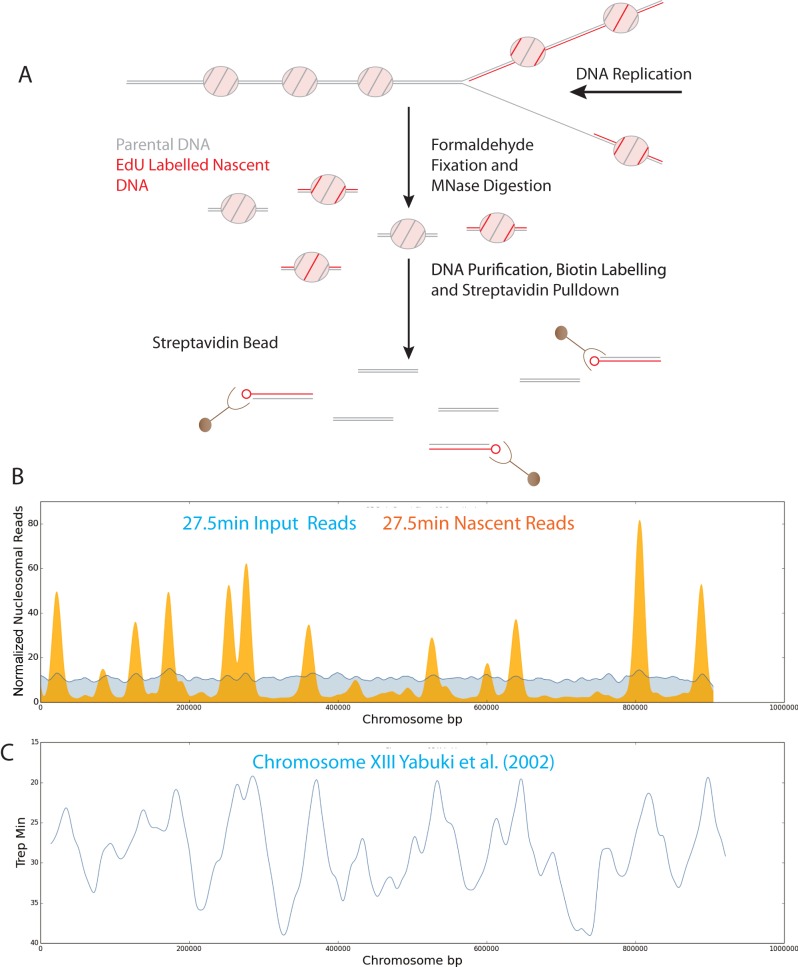
A system for isolation of nascent chromatin by EdU labelling of newly replicated DNA. (**A**) Schematic illustration of the EdU approach for isolation of nascent nucleosomal DNA. (**B**) Reads for replicating (nascent: orange) and unreplicated (input: blue) nucleosomal DNA per bp along chromosome 13 for an early S-phase time point, 27.5 min post-release from G1 arrest. (**C**) Replication profiles from previously annotated origins of replication for chromosome 13 identified by S-phase copy number (43).

The thymidine analogue 5-ethynyl-2′-deoxy-uridine (EdU) differs from thymidine only at the 5′ position and is incorporated by DNA polymerase in place of thymidine (39). Following incorporation into DNA, EdU can be coupled to biotinylated azide which provides a means of affinity purification (Figure 1A). To ensure that EdU was available for rapid incorporation we used a strain in which five copies of the herpes simplex thymidine kinase were expressed from GDP1 promoters (40) and the human equilabrative transporter 1 (ENT1) gene was expressed from the ADH1 promoter (41,42). Fluorescent labelling of EdU was used to assess the rate at which it gets incorporated into cells. A progressive increase in the number of cells with fluorescent foci was observed following incubation of an asynchronous culture with EdU between 5 and 60 min (Supplementary Figure S1A). This indicates that the time taken for EdU to enter cells and reach concentrations comparable with the endogenous pool of Thymidine is less than 5 min as foci will only be detected by microscopy once sizable tracts of EdU have been incorporated.

To provide a means of isolating chromatin assembled on recently replicated DNA, cultures were released from G1 arrest into media containing EdU. Chromatin was prepared from cultures at various time points and streptavidin beads used to purify replicated chromatin from the total input chromatin at each time point. When the distribution of nucleosomes on recently replicated DNA was plotted across chromosome XIII, reads were found to be highly enriched (c20-fold) and tightly distributed surrounding replication origins (43) 27.5 min following release from G1 arrest (Figure 1B and C). At later time points the enrichment at origins reduces and spreads away from origins consistent with the replication of the majority of the genome between 25 and 60 min following release from G1 arrest (Supplementary Figure S1B).

**Figure 2.**
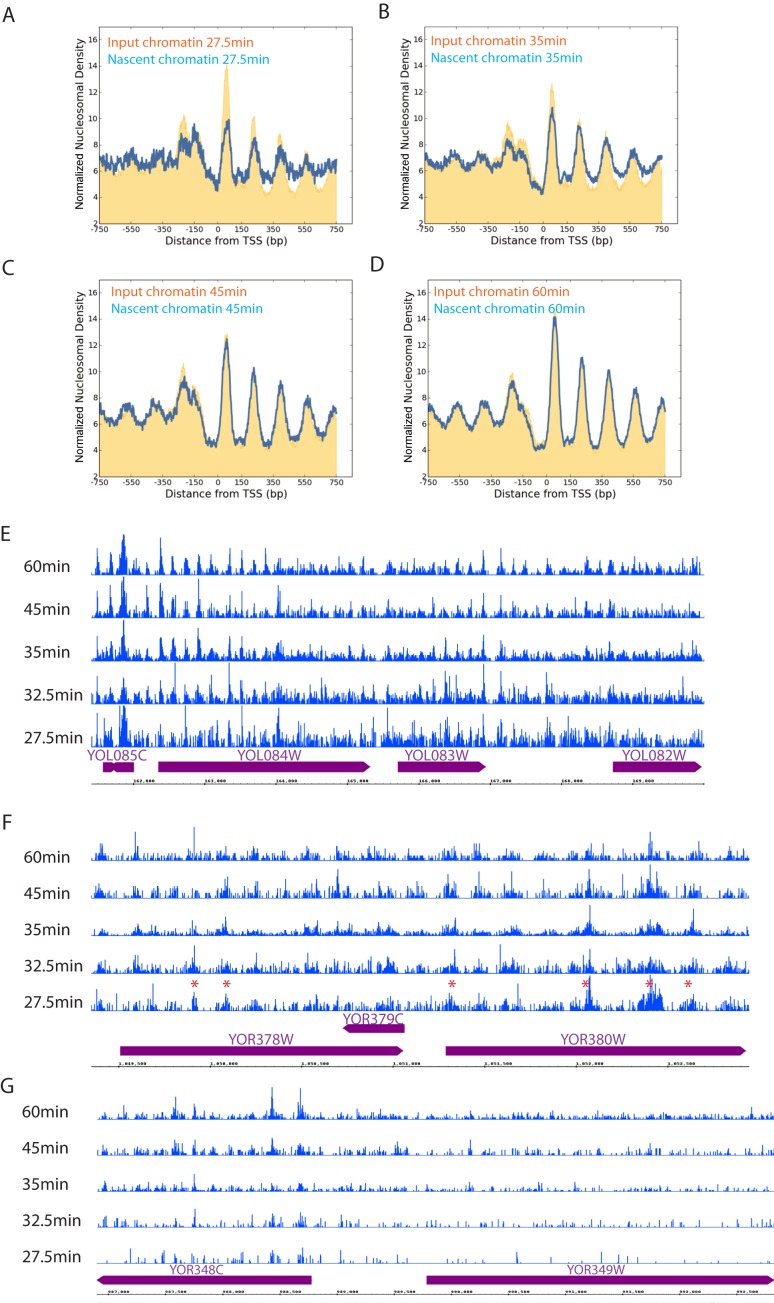
Characterization of nascent chromatin by EdU labelling of newly replicated DNA. Normalized frequency of nucleosome dyads aligned to the TSS of all genes (*n* = 5015) at the indicated time points following release from G1 arrest. The distribution of replicated fragments (nascent: blue) isolated by affinity purification of EdU labelled fragments is shown in comparison to the total chromatin isolated prior to pull down (input: orange) (**A**) 27.5, (**B**) 35, (**C**) 45, (**D**) 60 min following release from α factor arrest. Reads from EdU enriched chromatin isolated at the time points indicated following G1 arrest are shown across individual loci in (**E** and **F**). Across the locus shown in (**E**) many chromatin features are distinguishable at 27.5 min and the greatest maturation occurs between 27.5 and 32.5 min consistent with what is observed in the average profile of all genes. (**F**) Shows a locus at which many nucleosomes are less well defined and some chromatin features (indicated with a red asterisk) are detectable at the earliest time point and do not change or disperse over the time course. (**G**) This region is replicated later and as a result is depleted for reads isolated from early S-phase chromatin.

When nucleosomal reads were aligned with respect to promoters, it was notable that the amplitude of the nucleosomal oscillation was less pronounced than that observed in input chromatin (Figure 2A). Over subsequent time points promoter based nucleosome organization is restored to the state observed in input material (Figure 2A–D). This indicates that it is possible to monitor the re-establishment of chromatin organization in the minutes following replication. In order to investigate whether the maturation observed at all genes averaged was also observed at individual loci, the distribution of reads was plotted across selected loci. At regions close to origins where read depth at the early time points is high, nucleosomal features were apparent at the earliest time point and are often observed to become better defined at a rate consistent with the average at all genes (Figure 2E). In some cases, rates of maturation differed from the genome average, and for example appear to be established at the earliest time point and either decayed or remain unchanged (Figure 2F). Nascent chromatin from the early stages of replication was subject to greater amplification than used in conventional MNase-Seq reactions. This may contribute to the sporadic distribution of reads distant from replication origins (Figure 2G). The relatively disordered nature of nascent chromatin complicated the use of nucleosome calling algorithms and clustering to identify cohorts of genes that mature at similar rates.

### The kinetics of chromatin organization

**Figure 3.**
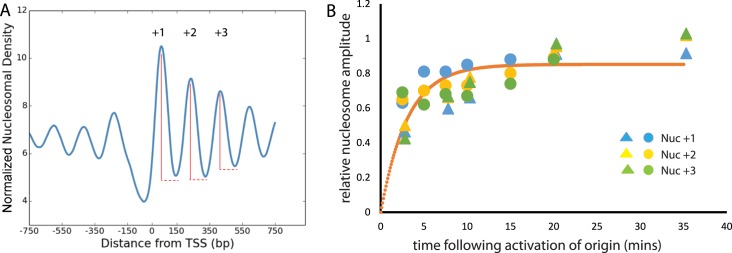
Kinetics of nucleosome organization. The depth of the oscillation in nucleosomal read depth was determined for the +1, +2 and +3 nucleosomes as indicated in (**A**). The oscillation depth in nascent chromatin at nuc +1 (blue), nuc +2 (yellow), nuc +3 (green) was then expressed as a fraction of that observed in the input chromatin for two repeats of an EdU time course at the time points indicated (**B**). The time values were calculated based on the distribution of replicated fragments observed at origins multiplied by the rate of elongation for DNA polymerase, 1.6 kb/min (17). Time points for two biological repeats are shown as circles and triangles. A fit to the first order rate equation, y = Ae^−(kt)^ is shown (orange) which allows estimation of the half time for nucleosome positioning as 2.1 min. The residual, *R*^2^, for this fit is 0.48.

Budding yeast have defined origins of replication, by definition the early stages of replication take place close to origins. The profile of reads surrounding origins allows the mean length of DNA replicated to be estimated within the vicinity of each isolated origin. The total length replicated at the 27.5 min time point typically ranges from 0 to 33 kb. Although, the base of the peak flanking many replication origins is ∼33 kb, the majority of the reads flanking each origin are considerably shorter. This arises from the fact that origin firing is stochastic (31) and as a result at later time points additional origins fire in different cells, but these have time to replicate progressively shorter regions. The distance from one side of an origin required to account for 50% of the read depth was calculated as 4500 ± 600 bp. This means that on average DNA polymerase has travelled 4500 kb at this time point. As the rate of DNA replication has been measured as 1.6 kb/min (17) this means that on average within the 27.5 min sample we can assume DNA had been replicated for 2.8 min. In addition, we can measure the extent to which chromatin is organized for nucleosomes at different positions within the coding region. This was achieved by measuring the amplitude of the nucleosomal oscillation (Figure 3A) in nascent chromatin as a fraction of that in the input chromatin for different time points. Relative nucleosome organization could then be plotted against the time following replication calculated with reference to the length distribution of fragments surrounding origins (Figure 3B). A fit of the data points to the rate equation for a first order reaction enables the half time for nucleosome organization to be estimated as 2.1 min.

### Nucleosomes are restored at replication origins within minutes of replication

The timing with which chromatin is restored is short, ∼2 min, in comparison to the half-time for transcription of yeast genes, 8 min (44). This raises the question, does the alignment of nucleosomes with promoters require transcription? One way of investigating this further is to study the organization of chromatin at cohorts of genes that are likely or unlikely to be expressed during the period of EdU labelling. To do this cohorts of genes were selected based on expression during the cell cycle (45). Nascent chromatin for genes expressed in G1 or S-phase was disordered at the 27.5 min time point (Supplementary Figure S2A and C). However, by 35 min from release from G1 arrest nucleosomes had adopted a more similar organization at genes expressed in S-phase in comparison to genes expressed in G1 (Supplementary Figure S2B and D). Little effect was observed if the maturation of chromatin was compared for genes expressed at high and low levels in asynchronous cultures (Supplementary Figure S2E–H). The stronger initial alignment of nucleosomes with genes expressed during S-phase could result from the coupling of ATP-dependent nucleosome spacing with transcriptional elongation. Alternatively, genes expressed in S-phase may have higher occupancy of bound transcription factors capable of acting as a reference point from which nucleosomal arrays can be established. Distinguishing between these explanations could be assisted by studying alignment of nucleosomes to a feature not involved in transcription.

**Figure 4.**
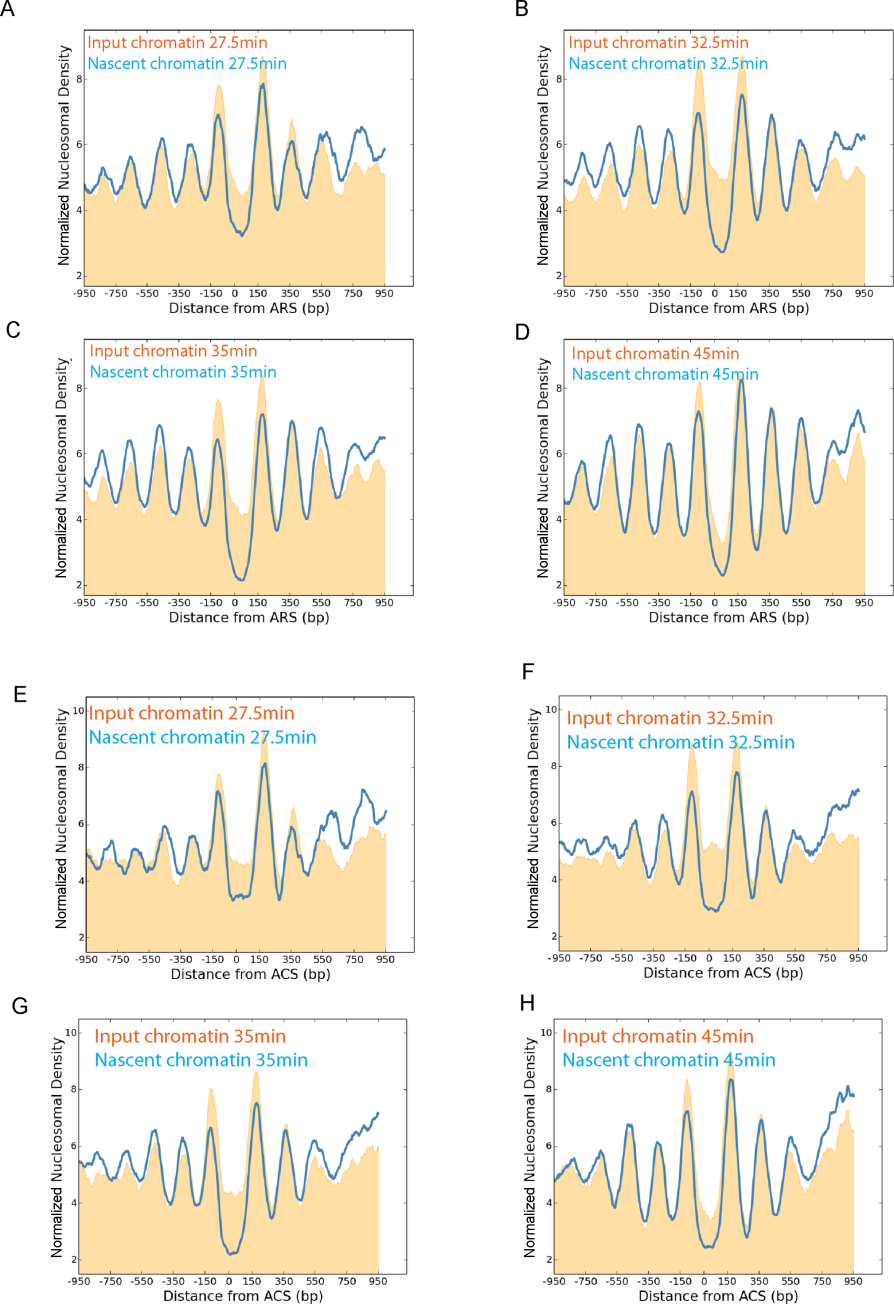
Chromatin maturation at origins of DNA replication. Chromatin from the EdU enrichment time course was plotted with respect to 205 replication origins (46). Nascent (blue) and input chromatin (orange) are plotted 27.5 (**A**), 32.5 (**B**), 35 (**C**) and 45 min (**D**) following release from G1 arrest. The +2 and +3 Nucleosomes are significantly ordered at the first time point and this improves over the following minutes. As many replication origins are located adjacent to transcribed genes, the same analysis was performed with 127 replication origins for which no TSS was present within 500 bp of the origin (**E–H**). Nucleosomes are not as precisely aligned to TSS-free origins in comparison to all origins (Compare input chromatin A–D to that of E–H). In particular the nucleosome depleted region at origins is poorly defined in early S-phase. Organization of the +2 and +3 nucleosomes at replication origins with no adjacent TSS mature at a similar rate to that observed at all origins.

Within the yeast genome it is known that nucleosomes are also aligned to replication origins (46). Alignment of nascent nucleosomes to replication origins shows that nucleosomes are substantially aligned with replication origins at the 27.5 min time point (Figure 4A). By 32.5 min the +2 and +3 nucleosomes are fully organized which is consistent with the half-time observed for chromatin restoration at promoters. The magnitude of the +1 nucleosome varies during S-phase perhaps reflecting changes to accessibility at origins during S-phase. Replication origins are often located close to promoters, so a subset of replication origins with no promoter located within 500 bp was also studied (Figure 4 E–H). At these 127 origins, positioning of the +2 and +3 nucleosomes was also re-established by 32.5 min following release from G1 arrest. This provides additional evidence that the realignment and spacing of nucleosomes does not require transcription.

### Defects in chromatin assembly result in disruption and delay in the organization of nascent chromatin

**Figure 5.**
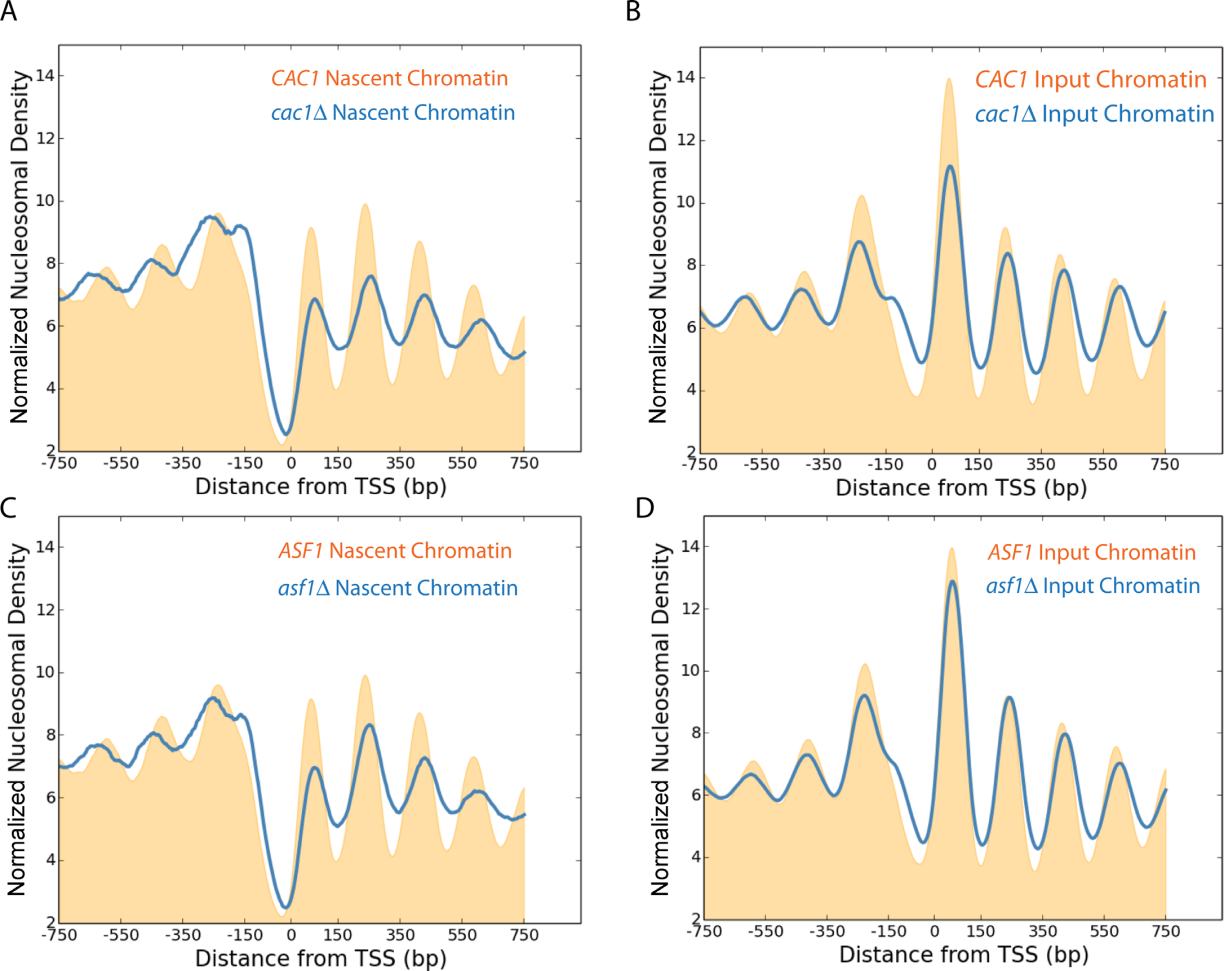
Loss of histone chaperones perturbs nascent chromatin organization. Normalized frequency of nascent nucleosomal dyads aligned to the TSS of all genes (*n* = 5015) in wild-type (orange), *cac1*Δ (blue) (**A**) and *asf1*Δ (blue) (**C**) deficient strains, 10 min following addition of EdU to an asynchronously growing culture. The normalized frequency of input chromatin prior to enrichment for newly replicated fragments for wild-type, *cac1*Δ (**B**) and *asf1*Δ strains (**D**).

It is known that histone chaperones such as Asf1 and Caf1 assist in the delivery and assembly of nucleosomes on newly replicated chromatin (47–50). The chromatin from asynchronous cultures of strains mutated for these chaperones show defects to nucleosome positioning of promoter distal nuclesosomes (51). We next investigated the effect mutations to these chaperones had on nascent nucleosome organization. Differences observed include a reduction in the amplitude of the nucleosome oscillation, a reduction in the occupancy of the +1 nucleosome and changes to the positioning of nucleosomes (Figure 5A and C). These changes were less prominent in mature chromatin (Figure 5B and D).

### Reduced histone supply results in increased inter-nucleosome spacing in nascent chromatin

The *cac1* mutant is especially interesting as in this strain it has been shown that fewer nucleosomes are deposited on replicated DNA in strains mutant for components of the CAF1 complex (52). This provides an opportunity to investigate the effect of nucleosome depletion during the course of chromatin organization. We found that the combination of growth in the presence of EdU and the *cac1* mutation resulted in substantial checkpoint activation. Prolonged exposure to EdU has previously been observed to activate DNA damage checkpoints (53,54) and in combination with mutation of *CAC1* progression through S-phase was severely disrupted, making it impossible to study the maturation of chromatin in this mutant using the EdU approach.

**Figure 6.**
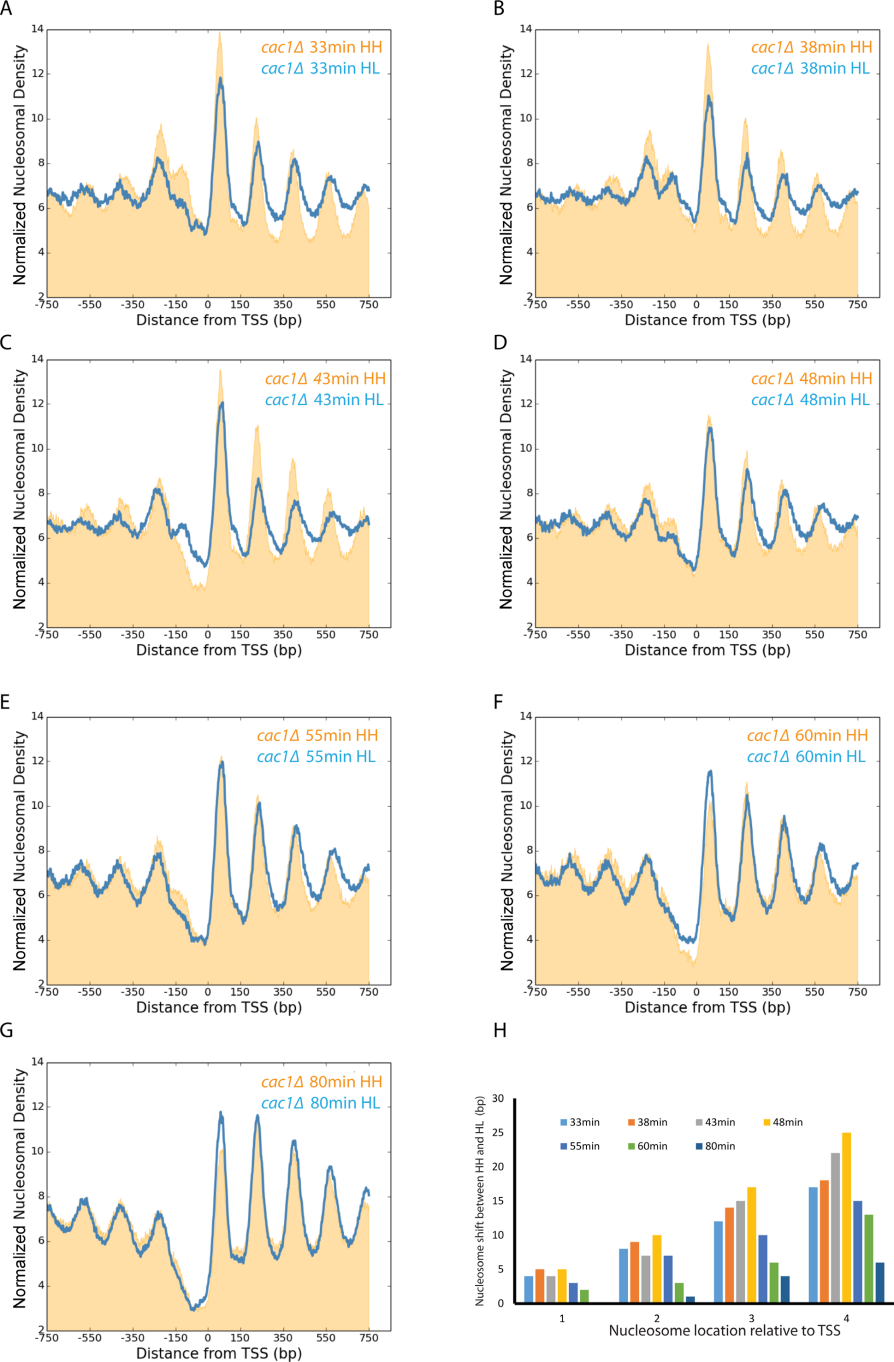
Alteration of nucleosome spacing in nascent chromatin in the absence of Cac1. Isolation of nascent DNA by isotope labelling and caesium chloride gradient density separation provides a means to track the relatively slow maturation of chromatin in a *cac1*Δ mutant. Normalized frequency of nucleosomal dyads aligned to the TSS for replicated HL (blue) and unreplciated HH (orange) labelled nucleosomal fragments isolated 33 (**A**), 38 (**B**), 43 (**C**), 48 (**D**), 55 (**E**), 60 (**F**) and 80 min (**G**) following release from G1 arrest. Quantitation of the 3′ shift in the average nucleosome location for +1, +2, +3 and +4 nucleosomes for each time point is shown in (**H**).

**Figure 7.**
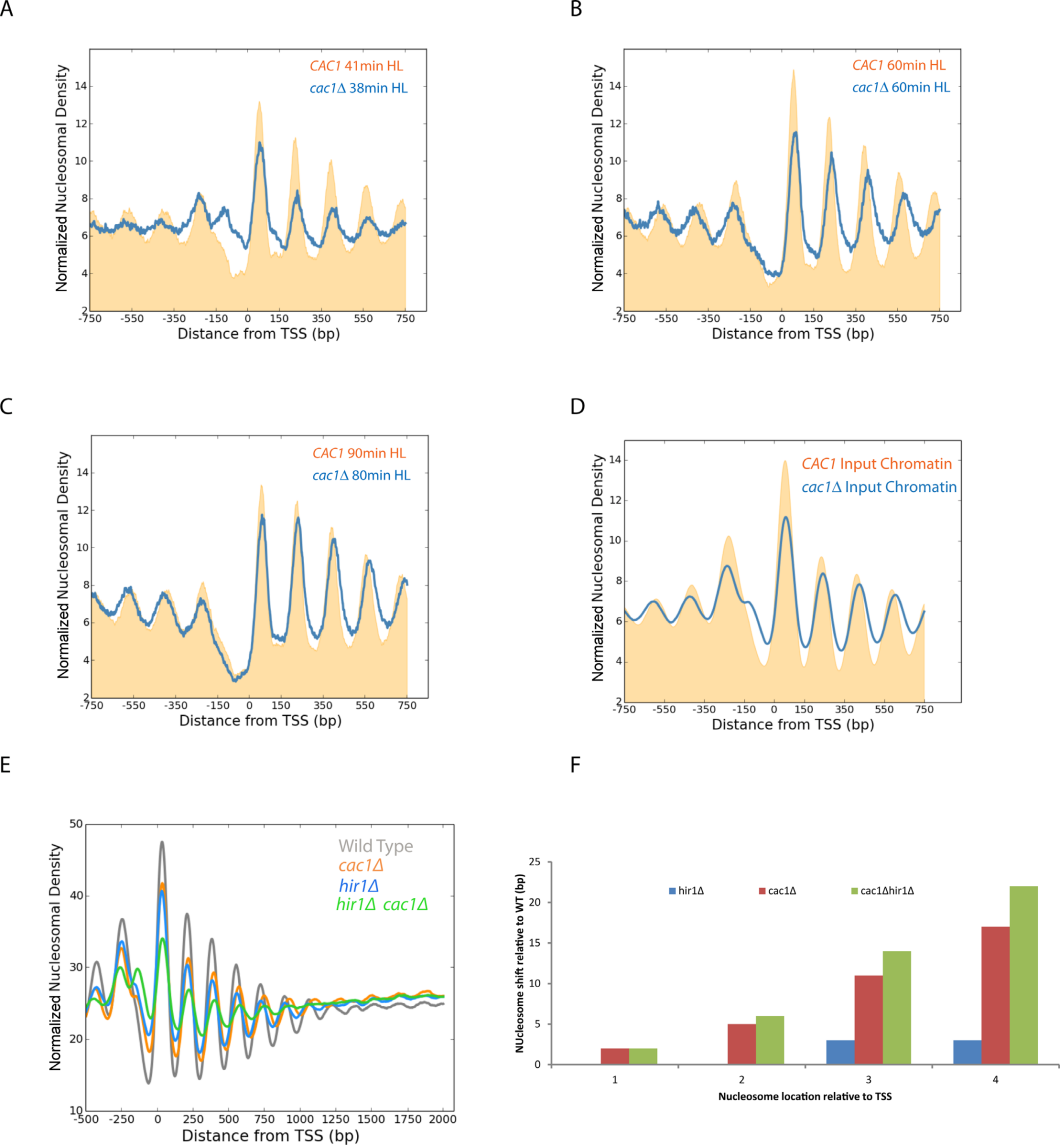
The defect to nucleosome spacing in the absence of Cac1 is restored post-replication and enhanced in the absence of replication-independent histone turnover. Alignment of nucleosomal reads on nascent DNA to the TSS in wild-type (orange) and *cac1*Δ strains (blue) illustrates progressive accumulation of a spacing defect in mid S-phase (**A** and **B**). This is restored in late S-phase (**C**). The spacing defect in asynchronous total chromatin (**D**) is less than that observed in mid S-phase (A and B). Nucleosomal reads from asynchronous wild-type, *cac1*Δ, *hir1*Δ and *hir1*Δ*cac1*Δ strains were aligned to the TSS of all genes (*n* = 5015) (**E**). The nucleosome depleted region at promoters is partially filled in a *hir1*Δ*cac1*Δ strain (green) in comparison to *cac1*Δ (orange), *hir1*Δ (blue) and wild-type (grey). The defect to nucleosome spacing is quantified in (**F**). The defect is increased in the *hir1*Δ*cac1*Δ consistent with replication-independent histone turnover acting to restore nucleosome density on coding regions.

Instead, we used an alternative approach to separate replicated DNA fragments. This involved adaption of the classical isotope labelling approach (55) for separation of nucleosome length DNA fragments. This relies on the ability of CsCl gradients to resolve the difference in the mass of DNA fragments labelled on both strands with heavy isotopes of ^13^C and ^15^N from replicated DNA in which only one strand includes heavy isotopes (Supplementary Figure S3A). Importantly, this involves no chemical change to DNA that could contribute to replication stress. This approach has previously been used to monitor the progression of replication genome wide (34), but is typically applied to the separation of fragments that are kilobases in length. In order to achieve separation of smaller fragments we increased the mass difference achieved by isotope labelling through growth on D-glucose-^13^C_6_,1,2,3,4,5,6,6-d7. This sugar enables heavy labelling of both carbon and non-exchangeable hydrogen atoms. These atoms result in an increase in the mass difference from 13 to 18 Da per base. Using this approach in synchronized cultures, nascent nucleosomes are observed to be enriched flanking replication origins (Supplementary Figure S3B and C). The earliest time point at which we could isolate replicated DNA from wild-type strains using this approach was 33 min following G1 arrest, at which time nucleosomes were observed to be significantly promoter aligned and to become fully aligned over subsequent time points (Supplementary Figure S3D–G). Adoption of this approach with the *cac1* mutant showed that replication proceeds with similar timing to the *CAC1* parental strain (Supplementary Figure S4) as has been observed previously (56). Alignment of nucleosomal reads to the TSS over this time course reveals progressive organization of nucleosomes indicated by an increase in the amplitude of the nucleosomal oscillation (Figure 6A–G). Interestingly, we also observe shifts in the centres of the nucleosomal peaks in nascent HL chromatin compared to unreplicated HH chromatin for the same time points (Figure 6A–G). Quantitation of this defect indicates that it is greatest at the 48 min time point which corresponds to mid S-phase and decreases as chromatin matures at later time points (Figure 6H). In addition, the number of base pairs with which each nucleosome is shifted increases in increments of ∼5 bp for progressively more 3′ nucleosomes (Figure 6H). This is consistent with an increase in the spacing between nucleosomes on nascent DNA from 165 to ∼170 bp. A similar increase in the length of dinucleosomal fragments was also observed providing a direct measure of transiently increased inter-nucleosome spacing (Supplementary Figure S6C). Comparing the maturation of chromatin between the nascent chromatin in wild-type and *cac1Δ* mutant strains shows that the defect to nucleosome positioning is most pronounced at time points in mid S-phase (Figure 7A and B). In late S-phase nucleosome spacing is restored almost to that observed in the wild-type (Figure 7C). As a result it seems plausible that a subpopulation of cells in S-phase contribute to the smaller defect in spacing observed in asynchronous cultures (Figure 7D).

The changes to spacing observed in Figures 6 and 7 indicate that mutation of *CAC1* results in the establishment of a promoter-based chromatin architecture with increased nucleosome spacing. This altered chromatin is then converted to a form that is more similar to that observed in the wild-type. One possible explanation for this would be that as a result of the *cac1* mutation nucleosomes are assembled at reduced density. However over time the normal density of nucleosomes is restored over coding regions. One way in which this could occur is as a result of post-replicative redistribution of nucleosomes via replication-independent histone turnover. It is known that replication-independent histone is higher at some regions, such as promoters than it is on coding regions (57,58). Thus it is possible that replication independent turnover could act to redistribute nucleosomes from sites of high turnover to coding regions. As the HIR complex is required for replication-independent histone turnover at many sites (58), we investigated this by studying nucleosome organization in *hir1* mutants. Mutation of *HIR1* alone results in a reduction to the amplitude of the nucleosomal oscillation on coding regions, but little change in nucleosome spacing (Figure 7E). In a *hir1Δ cac1Δ* double mutant there is increased occupancy of histones within the nucleosome depleted region. This is consistent with Hir1 normally playing a role in removing nucleosomes from the nucleosome depleted region (NDR) in the absence of Cac1. The nucleosomal oscillation is dampened in the *hir1Δ cac1Δ* strain indicating that nucleosomes are not spaced as effectively in this mutant. In addition the residual promoter based nucleosomes show a defect which is increased in comparison to that observed in the *cac1Δ* mutant. This is consistent with the idea that replication-independent histone turnover acts to restore nucleosome density and as a consequence nucleosome spacing over coding regions.

## DISCUSSION

The EdU-based affinity purification approach described here provides a means to quantitatively asses the realignment of nucleosomes with promoters genome wide. The system relies on the presence of defined origins of replication in budding yeast and the use of synchronized cultures. A limitation is that the timing with which individual replication origins fire is stochastic with individual origins initiating over a distribution of times (31). We address this by calculating timing based on the lengths of DNA fragments replicated at time points following release from arrest. This enables us to estimate the half time for reassembly of a promoter based chromatin architecture as ∼2 min. This time scale is also consistent with the data we obtained using isotope labelling. The enrichment for nascent chromatin was c6-fold using the CsCl approach in comparison to 20-fold using EdU meaning that we could not enrich for chromatin at very early time points. However, promoter based chromatin was largely (c90%) re-established at the earliest time point corresponding to 4.9 min post-replication (Supplementary Figure S3D). We have also isolated chromatin from asynchronous cultures following incubation with EdU for times as short as 5 min (Supplementary Figure S5). Using this approach, DNA is labelled at all distances from replication origins so we cannot use DNA fragment lengths to infer timing. The time taken for EdU to outcompete the intracellular pool of thymidine is not known, but is <5 min based upon detection of EdU tracts by microscopy (Supplementary Figure S1A). This means that the observation of c80% chromatin organization after 5 min could have occurred <5 min following replication but not more. Using all three approaches we observe that promoter-based chromatin is restored to between 70 and 90% of the level observed within native chromatin 5 min post-replication. Isolation of chromatin from earlier time points requires greater amplification. In our experience, the resulting data was not suited for high resolution nucleosome mapping, even after averaging for many genes.

Rapid chromatin reorganization post-replication is consistent with previous observations of chromatin reassembly behind replication forks within seconds (13–16). The initial deposition of histones is likely to occur so rapidly that we do not detect a substantially nucleosome free state. The read length distribution we observe in nascent chromatin shows a strong peak in the 165 bp size range consistent with the assembly of canonical nucleosomes. However, our fragment amplification was not tuned to identify subnucleosomal species that have been observed *in vitro* (59,60). Following the initial steps in nucleosome assembly, we find that nucleosomes are aligned to promoters over the following minutes. One previous study reported that three nucleosomes within the rDNA intragenic region are repositioned to the locations observed in asynchronous cells within a few seconds (18). This is considerably faster than we have observed here. It is possible that positioning of nucleosomes on the 5S intragenic regions is unusual in that is more strongly influenced by the underlying DNA sequence than is the case for most coding region nucleosomes. Supporting this, a DNA sequence that partially overlaps this locus has been observed to position nucleosomes similarly *in vivo* and *in vitro* (61). Rapid alignment of chromatin with promoters is also consistent with the alignment of Okazaki fragments with nucleosome dyads (62). Our study provides a more direct measurement of timing as in this approach Okazaki fragments are harvested typically 2.5 h after depletion of DNA ligase (62) and there is potential for the positions of nicks to change as a result of fragment maturation during this time (63).

Re-establishment of a promoter-based chromatin architecture over 2 min is fast in comparison to the half time for transcription of yeast genes, 8 min (44). This suggests promoter alignment does not require transcription which is further supported by the observation that nucleosome alignment occurs over a similar time course at replication origins where no coding transcription is anticipated. An attractive and simple model to explain the positioning of arrays of nucleosomes involves a barrier acting as a reference point from which nucleosomes are statistically positioned (64–66). A range of different DNA bound factors may be capable of acting in this way. For example, fortuitous binding of TFIIB has been observed to coincide with the establishment of promoter-like nucleosomal arrays (21). *In vitro* it has been observed that binding of lac repressor can act as a reference point for the phasing of nucleosome arrays (67). *In vivo* a number of factors including Tbf1, Reb1, Abf1 and Rsc3 are implicated in maintaining chromatin organization at promoters (51). The process of positioning could be facilitated by ATP-dependent nucleosome spacing enzymes such as Chd1 that are capable of redistributing nucleosomes to locations equidistant between neighbours (24,68–69). This provides a means by which the rapid alignment of nucleosomes with transcriptional start sites and replication origins could occur as a result of the rapid rebinding of DNA binding proteins which can then act as a reference point for positioning nucleosome arrays directed by remodelling enzymes. Changes in the distribution of strong DNA binding proteins capable of acting as reference points from which arrays are positioned could result in changes to the organization of nucleosomes at specific regions throughout the cell cycle or in response to environmental changes. This potentially provides an explanation for changes to chromatin at large cohorts of genes during the cell cycle and in response to metabolic changes (33,70) both of which are not necessarily linked to DNA replication. It is also worth mentioning that while our study has focused on chromatin organization post-replication, it is likely that there is a distinct transcription linked pathway that acts to restore chromatin following transit by RNA polymerases (28,71). If this is as rapid as replication coupled assembly, methods that can isolate chromatin in the minutes or seconds following transcription may be required to characterize this further.

Previous studies have shown that nucleosome spacing appears to be insensitive to a reduction in histone copy number (30,51,72–73). As consequence alternate models have been proposed in which linker DNA length is directly sensed during the course of nucleosome spacing reactions (30). Following depletion of CAF1 subunits it is known that histones are depleted and that nucleosome density is reduced in the total chromatin of asynchronous cultures (51,52). Given that CAF1 functions in chromatin assembly following replication, it is likely that histone supply is most severely compromised during the assembly following replication. We observe an increase in inter-nucleosome spacing from c165 to 170 bp in the nascent chromatin of a *cac1Δ* mutant yeast strain. This defect in spacing affects coding region nucleosomes and is most pronounced in mid S-phase when histone supply is likely to be most critical. In the minutes following replication, this extended spacing is restored to that observed in asynchronous cultures. Browsing through individual loci, the evidence for a change in nucleosome spacing is not as clear as in the data averaged for all genes (Supplementary Figure S6). A range of effects are observed. In many cases the dominant nucleosome positions are retained, but the pattern becomes less ordered around 48 min following release from G1 arrest (Supplementary Figure S6A) when the average defect is largest (Figure 6). In some cases shifts in positioning consistent with an increase in spacing are observed, but these are quite heterogeneous with some shifts appearing considerably larger than those observed on average (Supplementary Figure S6B). A major problem with using any single nucleosome positioning data to infer nucleosome spacing is that it is difficult to know which adjacent dyad locations observed in a population of cells are normally both occupied in the same cell. This is especially acute when nucleosome locations are not well defined as is the case for nascent chromatin. To address this, dinuclesomal fragments were sequenced. As dinucleosomal fragments encompass two nucleosomes and the intervening linker, they must be present on the same molecule. Assuming that the DNA protected by mononucleosomes remains constant, the change in dinucleosomal fragments reports directly on changes in linker length. A change in the mean length of dinucleosomal fragments is observed that is similar to the average change in mononuclesome positioning across genes (Supplementary Figure S6C). The size distribution of the HL dinucleosomes from the *cac1Δ* strain is quite broad at the early time points following release from arrest. This could result from the presence of nucleosomes deposited with variable spacing immediately following replication. In mid S-phase the distribution of dinucleosomal fragment lengths becomes better defined, but the most frequently observed lengths are just over 330 bp, 10 bp longer than observed in the unreplicated chromatin prepared from the same digests (Supplementary Figure S6C). As with the defect in mononucleosome spacing, this difference is reduced 80 min following replication. Minor differences between the data from mononucleosomes and dinucleosomes may reflect differences in the effects at coding regions (TSS aligned mononucleosomes) in comparison to all nucleosomes (dinucleosome data).

One of the most plausible explanations for the extension in linker length during S-phase is that reduced histone density during S-phase has an impact on statistically based spacing of nascent nucleosomes directed by ATP-dependent chromatin remodelling enzymes (64–66). Nucleosome spacing enzymes such as ISWI and Chd1 have many of the biophysical properties to accelerate a statistically based mechanism for nucleosome spacing. They can accelerate bidirectional nucleosome movement (74) and do so in a way that is sensitive to the length of DNA adjacent to nucleosomes (69,75–76). This sensitivity to linker DNA may act as a lower limit below which repositioning of adjacent nucleosomes is less efficient. Consistent with this different enzymes have been observed to establish arrays of nucleosomes with different periodicities *in vitro* (68) and changes to linker lengths are observed following changes to ionic conditions or incorporation of linker histones (68,77). Our observations are however more difficult reconcile with more recent reports using *in vitro* systems indicating that histone density does not to affect nucleosome spacing (30,78). It difficult to formally rule out the possibility that *cac1* mutations alter the expression or activity of specific remodelling enzymes. For example, it has recently been proposed that Isw1 acts to generate wider-spaced arrays of nucleosomes than Chd1 (79) and an increase in the relative contribution of Isw1 relative to Chd1 during S-phase could contribute to the observed effects. Further investigation will be required to resolve this.

A key question arising from the observation of altered spacing in the *cac1Δ* is how are nucleosomes restored to a periodicity more similar to that observed in wild-type strains in mature chromatin? One possible explanation is that histone depletion is unevenly distributed across genomes in post-replicative chromatin. There is evidence to support this as previous studies have noted reduced nucleosome occupancy following histone depletion at promoters, regions enriched for Htz1 and DNA sequences unfavourable for nucleosome formation (51,72–73). It is known that replication-independent histone turnover is more pronounced at specific genomic regions such as promoters and regions enriched for Htz1 while it is reduced at nucleosomes enriched for genic histone modifications (57,72). While replication-independent histone turnover acts to maintain an equilibrium between assembly and disassembly in wild-type cells this may be perturbed during conditions of histone depletion with the net effect of reducing histone occupancy at sites of high turnover and increasing it elsewhere. To investigate this further, we characterized nucleosome organization in which the Hir1 component of the HIRA complex has been mutated. This complex is required for replication-independent histone turnover at many sites in a range of species (58,80–82). Interestingly, it is required both for turnover at sites such as promoters and maintaining chromatin integrity over coding regions (83–85). We observe partial filling in of the NDR at promoters in *hir1Δ cac1Δ* double mutants consistent with a role for replication-independent turnover in influencing how a histone deficit is distributed across genes (Figure 7E). In addition, the defect in spacing is increased in asynchronous *hir1Δ cac1Δ* in comparison to *cac1Δ* (Figure 7F). This effect may be partially mitigated by the role the HIR complex plays in repressing histone gene expression outside of S-phase (86) as this would be anticipated to reduce rather than increase inter-nucleosome spacing. As a consequence we believe that replication-independent histone turnover mediated by HIRA and other factors has the potential to explain why histone depletion *in vivo* does not result in systematic changes in the nucleosomal repeat in asynchronous cultures.

The rapid re-establishment of chromatin means that the nucleosomal platform for gene expression is re-established prior to the partition of chromosomes into daughter cells. This potentially acts to maintain gene expression programs through cell divisions. However, it should be noted that while nucleosomes are rapidly reorganized, reestablishment of the distributions of certain histone modifications is rapid while for others it is delayed (4,87–88). One of the major consequences of a loss of nucleosome organization is increased intragenic transcription (28,51,84,89). Limiting the time during which chromatin is perturbed reduces the opportunity for potentially disruptive intragenic transcription. However, the disruption of chromatin during replication may also provide an opportunity for the reprogramming of expression. The 2 min half-time we have measured may balance these opposing requirements.

## Supplementary Material

SUPPLEMENTARY DATA
