# Supplementary material for: Establishment of a promoter-based chromatin architecture on recently replicated DNA can accommodate variable inter-nucleosome spacing
Source: Nucleic Acids Res. 2016 Apr 22;44(15):7189–203. doi: 10.1093/nar/gkw331 (PMC5009725; doi:10.1093/nar/gkw331)
Supplement: SUPPLEMENTARY DATA [file supp_44_15_7189__index.html]

Establishment of a promoter-based chromatin architecture on recently replicated DNA can accommodate variable inter-nucleosome spacing — SUPPLEMENTARY DATA 

# Establishment of a promoter-based chromatin architecture on recently replicated DNA can accommodate variable inter-nucleosome spacing

## SUPPLEMENTARY DATA

- SUPPLEMENTARY DATA
