# Supplementary material for: Establishment of a promoter-based chromatin architecture on recently replicated DNA can accommodate variable inter-nucleosome spacing
Source: Nucleic Acids Res. 2016 Apr 22;44(15):7189–203. doi: 10.1093/nar/gkw331 (PMC5009725; doi:10.1093/nar/gkw331)
Supplement: SUPPLEMENTARY DATA [file supp_gkw331_nar-00147-m-2016-File.zip › nar-00147-m-2016-File009.pdf]

Supplementary data 1 – .py (Python) file including the script used for alignment of reads to replication origins and promoters.

Strains used in this study

Strain	Genotype	Source
TT3532	*K5601 transformed with pT581/BsiW1 Mat-a GPD-TK (Thymidine Kinase)-Ura3 (5 copies), Padh1-hENT1::AUR1C, ade2-1, trp1-1, leu2-3, 112, his3-11,15, ura3*	(Saner, Karschau et al. 2013)
TOH 1386	*Mat a GPD-TK (Thymidine Kinase)-URA 3 (5 copies) Padh1- hENT1:: Aur1C trp1-1 leu2-3,112 his3-11, 15, Ade^{+} bar1Δ:: hphNT1*	RF This work
TOH 1389	*W303 MAT a trp1-1 can1-100 leu2-3,112 his3-11,15 Ura^{+} Ade^{+} bar1Δ::* hphNT1 *Gal+ psi+*	RF This work
TOH 1460	*Mat a GPD-TK (Thymidine Kinase)-URA 3 (5 copies) Padh1- hENT1:: Aur1C trp1-1 leu2-3,112 his3-11, 15, ade2-1, bar1Δ:: hphNT1 ,asf1Δ:: natNT2*	RF This work
TOH 1462	*Mat a GPD-TK (Thymidine Kinase)-URA 3 (5 copies) Padh1- hENT1:: Aur1C trp1-1 leu2-3,112 his3-11, 15 , ade2-1, bar1Δ:: hphNT1, cac1Δ:: natNT2*	RF This work
TOH 1521	*Mat a GPD-TK (Thymidine Kinase)-URA 3 (5 copies) Padh1- hENT1:: Aur1C trp1-1 leu2-3,112 his3-11, 15 , bar1Δ:: hphNT1, cac1Δ:: natNT2*	RF This work
TOH 1524	*Mat a GPD-TK (Thymidine Kinase)-URA 3 (5 copies) Padh1- hENT1:: Aur1C trp1-1*	RF This work

	leu2-3,112 his3-11, 15 , ade2-1, bar1Δ:: hphNT1, hir1Δ:: natNT2	
TOH 1525	*Mat a GPD-TK (Thymidine Kinase)-URA 3 (5 copies) Padh1- hENT1:: Aur1C trp1-1 leu2-3,112 his3-11, 15 , ade2-1, bar1Δ:: hphNT1, cac1Δ:: natNT2, hir1Δ:: TRP1*	RF This work

Supplementary Figure 1 – Labelling of nascent DNA by EdU incorporation.

A) An asynchronous yeast culture was incubated with EdU for the indicated times. Cells were fixed and click chemistry performed to attach Alexa Fluor 488 at sites of EdU incorporation. Bright field and fluorescence images are shown and indicate that the proportion of cells with tracts of EdU long enough for detection by microscopy are visible in 0%, 5%, 18% and 60% of cells after 3, 5, 10 and 60 min incubation with EdU.

B) Normalised, twice smoothed (10,000 bp moving average) nascent reads for the time points indicated were plotted as a function of chromosomal coordinate (bp) along chromosome 13. At earlier S-phase time points there is an enrichment of nascent reads proximal to origins of replication. Reads are redistributed across chromosome 13 as S-phase progresses.

C) The distribution of nascent EdU labelled (orange) and input (blue) nucleosomal reads isolated from an asynchronous culture is plotted as a function of chromosomal coordinate (bp) along chromosome 13. The reads are evenly distributed across the chromosome consistent with incorporation at sites of replication randomly distributed across the chromosome.

Supplementary Figure 2 - Interplay between gene expression and chromatin maturation.

Cohorts of 162 genes expressed in S-phase **A,B)**, or 212 genes expressed in G1 **C,D)**, were identified using the data of (Rowicka, Kudlicki et al. 2007). Nascent chromatin isolated after 27.5 min **A,C)**, or 35 min **B,D)**, following release from G1 arrest was aligned to the TSS. In all cases significant alignment of nucleosomes with the TSS is observed after 35 min. 27.5 min from G1 arrest alignment of nucleosomes is weaker at genes expressed in G1. The top 10% of highly expressed genes **D, E)** and bottom 10% of genes expressed at lowest levels **F, G)** were identified based upon the data of (Lee, Tillo et al. 2007). Nascent and input chromatin from EdU enrichment was plotted with respect to the TSS.

Supplementary Figure 3- Isolation and characterisation of nascent chromatin by stable isotope labelling and CsCl gradient ultracentrifugation.

A) Schematic for isolation of nascent nucleosomal DNA **B**) Normalised, twice smoothed (10,000 bp moving average) nucleosomal reads for replicating Heavy Light (HL-orange) and unreplicated Heavy Heavy (HH-blue) DNA along chromosome 13 for an early S-phase time point, 33min post release from G1 arrest. **C)** Replication profiles from previously annotated origins of replication for chromosome 13 identified by S-phase copy number (Yabuki, Terashima et al. 2002). **D-G)** Normalised frequency of nucleosome dyads aligned to the TSS of all genes (n=5015) at the time points indicated

following release from G1 arrest. Replicated (HL-blue) and non-replicated (HH-orange) DNA is shown for each time point.

Supplementary Figure 4 – Analysis of DNA replication progression from chromatin in wild type and *cac1* mutant strains using stable isotope labelling.

Normalised, twice smoothed (10,000 bp moving average) nascent (HL labelled) reads for S-phase time points were plotted as a function of chromosomal coordinate (bp) along chromosome 13 in **A)** wild type and **B)** *cac1Δ* strains. Distributions are plotted for nucleosomal DNA isolated at different times following release from G1 arrest as indicated. Reads are redistributed evenly across chromosome 13 as S-phase progresses in both replication profiles.

Supplementary Figure 5 - Nucleosome positioning in nascent chromatin isolated from asynchronous cultures. The normalised frequency of nucleosome dyads aligned to the TSS of all genes **A)** 5, **B)** 10 and **C)** 50 minutes following addition of EdU to an asynchronously growing culture. Replicated (nascent – blue) and non-replicating DNA (input – orange) is shown for each time point. Nucleosome organisation improves for the samples incubated with EdU for longer time periods.

Supplementary Figure 6 – Direct measurement of changes in nucleosome spacing. Data from the time course experiment presented in Figure 6 was plotted at selected loci using Integrated Genome Browser, version: 8.1.11 (Nicol, Helt et al. 2009). **A)** At YOR196C and YOR197W, several prominent nucleosomes are retained through the time course. However, around the 48 min time point the nucleosomal reads are more evenly distributed making it difficult to assess nucleosome spacing. **B)** At YNRO32W, a subset of nucleosomes appears to be positioned out of phase during mid S-phase (indicated with red asterix, *). **C)** To more directly assess nucleosome spacing the dinucleosomal band was excised from a gel of the MNase digested chromatin as prepared for Figure 6 and sequenced. The length distributions of the dinucleosomal fragments are plotted for the nascent (HL-blue) and unreplicated (HH-orange) chromatin for the indicated time points. The sizes of the dinucleosomal maxima are indicated. The size of the dinucleosomal fragments increases in mid S-phase consistent with the transient change in nucleosome spacing observed in Figure 6.

References

Lee, W., D. Tillo, N. Bray, R. H. Morse, R. W. Davis, T. R. Hughes and C. Nislow (2007). "A high-resolution atlas of nucleosome occupancy in yeast." Nature Genetics **39**(10): 1235-1244.

Nicol, J. W., G. A. Helt, S. G. Blanchard, A. Raja and A. E. Loraine (2009). "The Integrated Genome Browser: free software for distribution and exploration of genome-scale datasets." Bioinformatics **25**(20): 2730-2731.

Rowicka, M., A. Kudlicki, B. P. Tu and Z. Otwinowski (2007). "High-resolution timing of cell cycle-regulated gene expression." Proc Natl Acad Sci U S A **104**(43): 16892-16897.

Saner, N., J. Karschau, T. Natsume, M. Gierlinski, R. Retkute, M. Hawkins, C. A. Nieduszynski, J. J. Blow, A. P. de Moura and T. U. Tanaka (2013). "Stochastic association of neighboring replicons creates replication factories in budding yeast." J Cell Biol **202**(7): 1001-1012.
Yabuki, N., H. Terashima and K. Kitada (2002). "Mapping of early firing origins on a replication profile of budding yeast." Genes Cells **7**(8): 781-789.
