# Supplementary material for: Establishment of a promoter-based chromatin architecture on recently replicated DNA can accommodate variable inter-nucleosome spacing
Source: Nucleic Acids Res. 2016 Apr 22;44(15):7189–203. doi: 10.1093/nar/gkw331 (PMC5009725; doi:10.1093/nar/gkw331)
Supplement: SUPPLEMENTARY DATA [file supp_gkw331_nar-00147-m-2016-File.zip › nar-00147-m-2016-File016.pdf]

No.	Filename	Used in figure(s)	Mononucleosomal Read Length	Reads
**1**	EdU_Synch_Input_275min	1, 2 ,3, 4 ,S1 ,S2	170	7807061
**2**	EdU_Synch_nascent_275min	1, 2 ,3, 4 ,S1, S2	169	23785208
**3**	EdU_Synch_Input_325min	2 ,3, 4,	168	7364516
**4**	EdU_Synch_nascent_325min	2 ,3, 4, S1	167	23204093
**5**	EdU_Synch_Input_35min	2 ,3, 4 , S2	168	7167674
**6**	EdU_Synch_nascent_35min	2 ,3, 4 ,S1, S2	169	27984712
**7**	EdU_Synch_Input_45min	2 ,3, 4	170	6683387
**8**	EdU_Synch_nascent_45min	2 ,3, 4, S1	170	22264186
**9**	EdU_Synch_Input_60min	2 ,3	168	7012153
**10**	EdU_Synch_nascent_60min	2 ,3, S1	169	22926824
**11**	EdU_Synch_Input_275min_bioreplicate	3	159	10099750
**12**	EdU_Synch_nascent_275min_bioreplicate	3	168	32041662
**13**	EdU_Synch_Input_30min_bioreplicate	3	159	7611866
**14**	EdU_Synch_nascent_30min_bioreplicate	3	169	28502186
**15**	EdU_Synch_Input_325min_bioreplicate	3	159	9118408
**16**	EdU_Synch_nascent_325min_bioreplicate	3	167	24370448
**17**	EdU_Synch_Input_35min_bioreplicate	3	157	76521284
**18**	EdU_Synch_nascent_35min_bioreplicate	3	159	51840154
**19**	EdU_Synch_Input_40min_bioreplicate	3	158	73112718
**20**	EdU_Synch_nascent_40min_bioreplicate	3	159	50645274
**21**	EdU_Synch_Input_45min_bioreplicate	3	157	88651036
**22**	EdU_Synch_nascent_45min_bioreplicate	3	159	52075736
**23**	WT_CsCl_G1_HH		164	34790607
**24**	WT_CsCl_G1_HL		161	36736894
**25**	WT_CsCl_33min_HH	S3	163	8558948
**26**	WT_CsCl_33min_HL	S3, S4	165	47707047
**27**	WT_CsCl_41min_HH	S3	162	12998204
**28**	WT_CsCl_41min_HL	7, S3, S4	166	54232004
**29**	WT_CsCl_60min_HH	S3	159	10883533
**30**	WT_CsCl_60min_HL	7, S3, S4	160	55788384
**31**	WT_CsCl_90min_HH	S3	159	12733851
**32**	WT_CsCl_90min_HL	7, S3, S4	160	63397063
**33**	WT_CsCl_G1_HH_bioreplicate		167	7708872
**34**	WT_CsCl_G1_HL_bioreplicate		164	40661512

**35**	WT_CsCl_33min_HH_bioreplicate		167	8469980
**36**	WT_CsCl_33min_HL_bioreplicate		167	31553378
**37**	WT_CsCl_41min_HH_bioreplicate		168	6370618
**38**	WT_CsCl_41min_HL_bioreplicate		167	34014538
**39**	WT_CsCl_60min_HH_bioreplicate		163	7340466
**40**	WT_CsCl_60min_HL_bioreplicate		166	35778300
**41**	WT_Async_EdU_Pulse_Input_5min	7, S1, S5	161	5595212
**42**	WT_Async_EdU_Pulse_nascent_5min	S1, S5	170	29455502
**43**	WT_Async_EdU_Pulse_Input_10min	S5	162	5789604
**44**	WT_Async_EdU_Pulse_nascent_10min	S5	162	36731264
**45**	WT_Async_EdU_Pulse_Input_50min	S5	161	5950174
**46**	WT_Async_EdU_Pulse_nascent_50min	S5	162	27758768
**47**	WT_Async_EdU_Pulse_Input_bioreplicate		169	16786974
**48**	WT_Async_EdU_Pulse_nascent_2min_bioreplicate		170	37922768
**49**	WT_Async_EdU_Pulse_nascent_5min_bioreplicate		170	32327936
**50**	WT_Async_EdU_Pulse_nascent_10min_bioreplicate	5	169	34317910
**51**	WT_Async_EdU_Pulse_nascent_50min_bioreplicate		169	38066752
**52**	WT_Async_EdU_Pulse_nascent_1hr30min_bioreplicate		169	33688236
**53**	Δasf1_Async_EdU_Pulse_control	5	162	55023366
**54**	Δasf1_Async_EdU_Pulse_nascent_1_high_reads	5	170	51566440
**55**	Δasf1_Async_EdU_Pulse_nascent_techreplicate2_high_reads		170	50076622
**56**	Δasf1_Async_EdU_Pulse_nascent_ techreplicate3_high_reads		170	42734982
**57**	Δasf1_Async_EdU_Pulse_control_bioreplicate1		168	17597929
**58**	Δasf1_Async_EdU_Pulse_nascent_bioreplicate1_high_reads		170	67532774
**59**	Δasf1_Async_EdU_Pulse_cont		168	15540686

	rol_bioreplicate2			
**60**	Δasf1_Async_EdU_Pulse_nascent_bioreplicate2_low_reads		183	23650204
**61**	Δasf1_Async_EdU_Pulse_control_bioreplicate3		168	6707569
**62**	Δasf1_Async_EdU_Pulse_nascent_bioreplicate3_low_reads		179	29309817
**63**	Δcac1_Async_EdU_Pulse_control		167	39405164
**64**	Δcac1_Async_EdU_Pulse_nascent_1 _high_reads	5	170	36689676
**65**	Δcac1_Async_EdU_Pulse_nascent_techreplicate2_high_reads		170	35488968
**66**	Δcac1_Async_EdU_Pulse_nascent_ techreplicate3_high_reads		170	25806866
**67**	Δcac1_Async_EdU_Pulse_control_bioreplicate1		167	18701647
**68**	Δcac1_Async_EdU_Pulse_nascent_bioreplicate1_low_reads		155	1312412
**69**	Δcac1_Async_EdU_Pulse_control_bioreplicate2		167	13133468
**70**	Δcac1_Async_EdU_Pulse_nascent_bioreplicate2_low_reads		159	9512504
**71**	Δcac1_Async_EdU_Pulse_control_bioreplicate3		168	14135825
**72**	Δcac1_Async_EdU_Pulse_nascent_bioreplicate3_ high_reads		166	19398644
**73**	Δcac1_Async_EdU_Pulse_control_bioreplicate4	5,7	167	17400264
**74**	Δcac1_CsCl_33min_HH_mononucleosome	6	167	12730156
**75**	Δcac1_CsCl_33min_HL_mononucleosome	6, S4, S6	169	50035525
**76**	Δcac1_CsCl_38min_HH_mononucleosome	6	168	6879117
**77**	Δcac1_CsCl_38min_HL_mononucleosome	6, 7, S4, S6	168	32182872
**78**	Δcac1_CsCl_43min_HH_mononucleosome	6	167	7454618
**79**	Δcac1_CsCl_43min_HL_mononucleosome	6, S4, S6	167	24011142
**80**	Δcac1_CsCl_48min_HH_mononucleosome	6	168	7128838
**81**	Δcac1_CsCl_48min_HL_mononucleosome	6, S4, S6	168	28367906
**82**	Δcac1_CsCl_55min_HH_mononucleosome	6	167	7507239
**83**	Δcac1_CsCl_55min_HL_mononucleosome	6, S4, S6	168	25924501

**84**	Δcac1_CsCl_60min_HH_mono nucleosome	6	168	6631763
**85**	Δcac1_CsCl_60min_HL_mono nucleosome	6, 7 , S4, S6	169	27505994
**86**	Δcac1_CsCl_80min_HH_mono nucleosome	6	160	7380217
**87**	Δcac1_CsCl_80min_HL_mono nucleosome	6, 7, S4, S6	166	27648588
**88**	Δcac1_CsCl_33min_HH_dinucl eosome	S6	Dinucleosomal	31465084
**89**	Δcac1_CsCl_33min_HL_dinucl eosome	S6	Dinucleosomal	31276640
**90**	Δcac1_CsCl_38min_HH_dinucl eosome	S6	Dinucleosomal	31007869
**91**	Δcac1_CsCl_38min_HL_dinucl eosome	S6	Dinucleosomal	28364605
**92**	Δcac1_CsCl_43min_HH_dinucl eosome	S6	Dinucleosomal	31827332
**93**	Δcac1_CsCl_43min_HL_dinucl eosome	S6	Dinucleosomal	27960159
**94**	Δcac1_CsCl_48min_HH_dinucl eosome	S6	Dinucleosomal	29885373
**95**	Δcac1_CsCl_48min_HL_dinucl eosome	S6	Dinucleosomal	28984478
**96**	Δcac1_CsCl_55min_HH_dinucl eosome	S6	Dinucleosomal	28498108
**97**	Δcac1_CsCl_55min_HL_dinucl eosome	S6	Dinucleosomal	24877012
**98**	Δcac1_CsCl_60min_HH_dinucl eosome	S6	Dinucleosomal	26750773
**99**	Δcac1_CsCl_60min_HL_dinucl eosome	S6	Dinucleosomal	24634102
**100**	Δcac1_CsCl_80min_HH_dinucl eosome	S6	Dinucleosomal	29802149
**101**	Δcac1_CsCl_80min_HL_dinucl eosome	S6	Dinucleosomal	22342117
**102**	Δcac1_CsCl_38min_HH_mono nucleosome_techreplicate		168	21992204
**103**	Δcac1_CsCl_38min_HL_mono nucleosome_techreplicate		170	124021052
**104**	Δcac1_CsCl_43min_HH_mono nucleosome_techreplicate		169	21209870
**105**	Δcac1_CsCl_43min_HL_mono nucleosome_techreplicate		133 ★	133672986
**106**	Δcac1_CsCl_55min_HH_mono nucleosome_techreplicate		168	22652596
**107**	Δcac1_CsCl_55min_HL_mono nucleosome_techreplicate		170	138030465
**108**	Δcac1_CsCl_60min_HH_mono nucleosome_techreplicate		168	22861963

**109**	Δcac1_CsCl_60min_HL_mononucleosome_techreplicate		170	119438003
**110**	Δcac1_CsCl_80min_HH_mononucleosome_techreplicate		161	21019517
**111**	Δcac1_CsCl_80min_HL_mononucleosome_techreplicate		166	132205124
**112**	Δcac1_CsCl_38min_HH_dinucleosome_techreplicate		Dinucleosomal	21115306
**113**	Δcac1_CsCl_38min_HL_dinucleosome_techreplicate		Dinucleosomal	19618665
**114**	Δcac1_CsCl_43min_HH_dinucleosome_techreplicate		Dinucleosomal	27096963
**115**	Δcac1_CsCl_55min_HH_dinucleosome_techreplicate		Dinucleosomal	21294495
**116**	Δcac1_CsCl_55min_HL_dinucleosome_techreplicate		Dinucleosomal	29782274
**117**	Δcac1_CsCl_60min_HH_dinucleosome_techreplicate		Dinucleosomal	25399842
**118**	Δcac1_CsCl_60min_HL_dinucleosome_techreplicate		Dinucleosomal	17984515
**119**	Δcac1_CsCl_80min_HH_dinucleosome_techreplicate		Dinucleosomal	32983938
**120**	Δcac1_CsCl_80min_HL_dinucleosome_techreplicate		Dinucleosomal	26250620
**121**	Δcac1_CsCl_33min_HH_mononucleosome_bioreplicate		167	45904815
**122**	Δcac1_CsCl_33min_HL_mononucleosome_bioreplicate		166	36490157
**123**	Δcac1_CsCl_38min_HH_mononucleosome_bioreplicate		165	41460790
**124**	Δcac1_CsCl_38min_HL_mononucleosome_bioreplicate		178	35730841
**125**	Δcac1_CsCl_43min_HH_mononucleosome_bioreplicate		170	35897429
**126**	Δcac1_CsCl_43min_HL_mononucleosome_bioreplicate		167	34441815
**127**	Δcac1_CsCl_48min_HH_mononucleosome_bioreplicate		165	38019311
**128**	Δcac1_CsCl_48min_HL_mononucleosome_bioreplicate		169	34518730
**129**	Δcac1_CsCl_55min_HH_mononucleosome_bioreplicate		165	35342203
**130**	Δcac1_CsCl_55min_HL_mononucleosome_bioreplicate		168	36023311
**131**	Δcac1_CsCl_60min_HH_mononucleosome_bioreplicate		165	39826390
**132**	Δcac1_CsCl_60min_HL_mononucleosome_bioreplicate		168	36450404
**134**	Δcac1_CsCl_80min_HH_mononucleosome_bioreplicate		159	40880723

**135**	Δcac1_CsCl_80min_HL_mononucleosome_bioreplicate		169	38089608
**136**	Δcac1_CsCl_33min_HH_dinucleosome_bioreplicate		Dinucleosomal	37462394
**137**	Δcac1_CsCl_33min_HL_dinucleosome_bioreplicate		Dinucleosomal	33312552
**138**	Δcac1_CsCl_38min_HH_dinucleosome_bioreplicate		Dinucleosomal	38825105
**139**	Δcac1_CsCl_38min_HL_dinucleosome_bioreplicate		Dinucleosomal	34367090
**140**	Δcac1_CsCl_43min_HH_dinucleosome_bioreplicate		Dinucleosomal	37752826
**141**	Δcac1_CsCl_43min_HL_dinucleosome_bioreplicate		Dinucleosomal	37608720
**142**	Δcac1_CsCl_48min_HH_dinucleosome_bioreplicate		Dinucleosomal	37770684
**143**	Δcac1_CsCl_48min_HL_dinucleosome_bioreplicate		Dinucleosomal	36963473
**144**	Δcac1_CsCl_55min_HH_dinucleosome_bioreplicate		Dinucleosomal	32957247
**145**	Δcac1_CsCl_55min_HL_dinucleosome_bioreplicate		Dinucleosomal	32983518
**146**	Δcac1_CsCl_60min_HH_dinucleosome_bioreplicate		Dinucleosomal	35067086
**147**	Δcac1_CsCl_60min_HL_dinucleosome_bioreplicate		Dinucleosomal	33234553
**148**	Δcac1_CsCl_80min_HH_dinucleosome_bioreplicate		Dinucleosomal	38794415
**149**	Δcac1_CsCl_80min_HL_dinucleosome_bioreplicate		Dinucleosomal	33694185
**150**	Δhir1_asynchronous	7	167	19682054
**151**	Δhir1_asynchronous_bioreplicate		167	14559026
**152**	Δcac1_Δhir1_asynchronous	7	167	18096211
**153**	Δcac1_Δhir1_asynchronous_bioreplicate		168	18337703

Supplementary Table 1

★ - Sample library required more rounds of PCR amplification and was considered a poor sample.
