# Supplementary figures and images for: Establishment of a promoter-based chromatin architecture on recently replicated DNA can accommodate variable inter-nucleosome spacing
Source: Nucleic Acids Res. 2016 Apr 22;44(15):7189–203. doi: 10.1093/nar/gkw331 (PMC5009725; doi:10.1093/nar/gkw331)

A

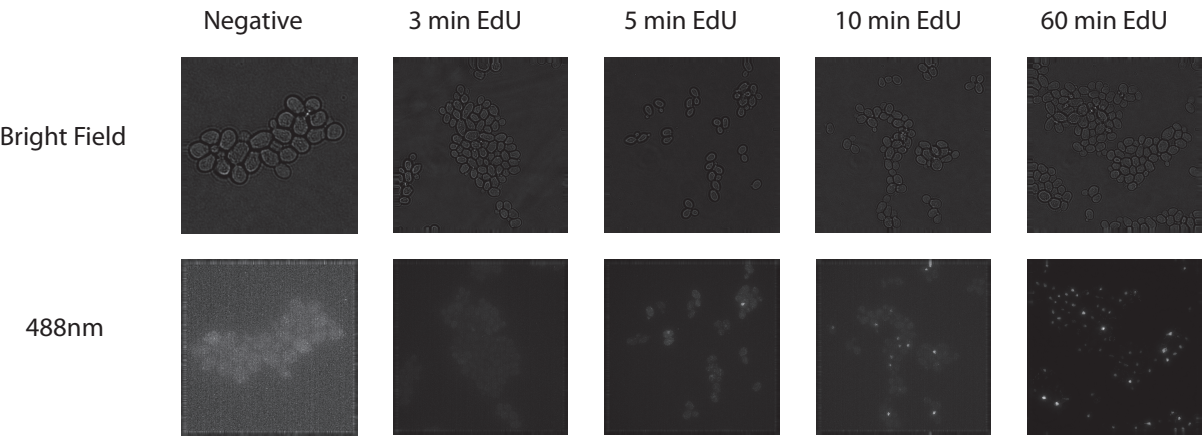

B

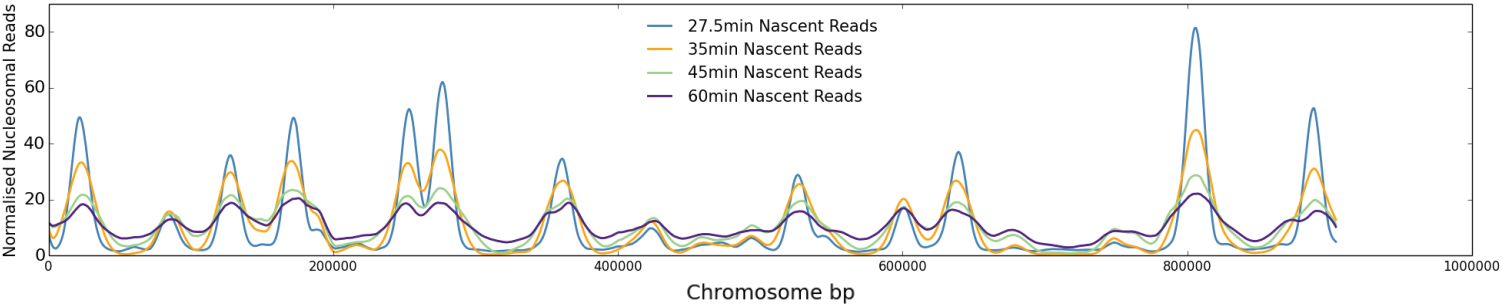

C

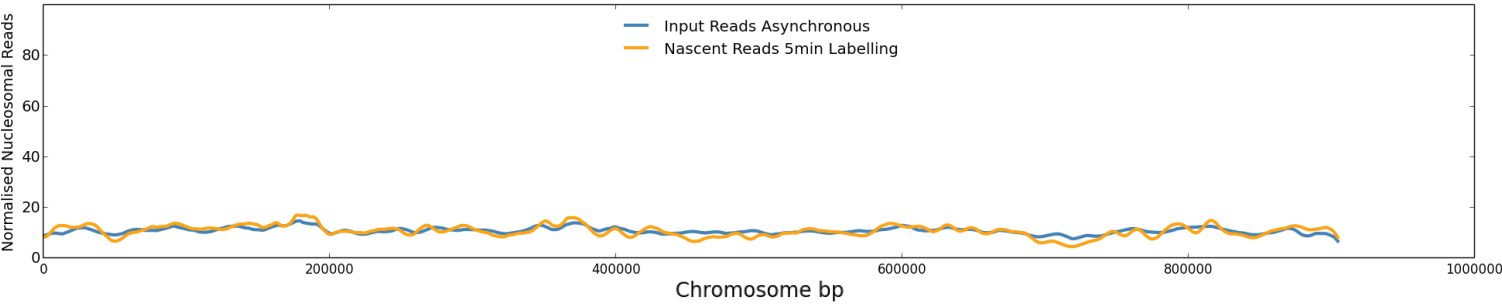

Supplement: SUPPLEMENTARY DATA [file supp_gkw331_nar-00147-m-2016-File.zip › nar-00147-m-2016-File010.pdf]

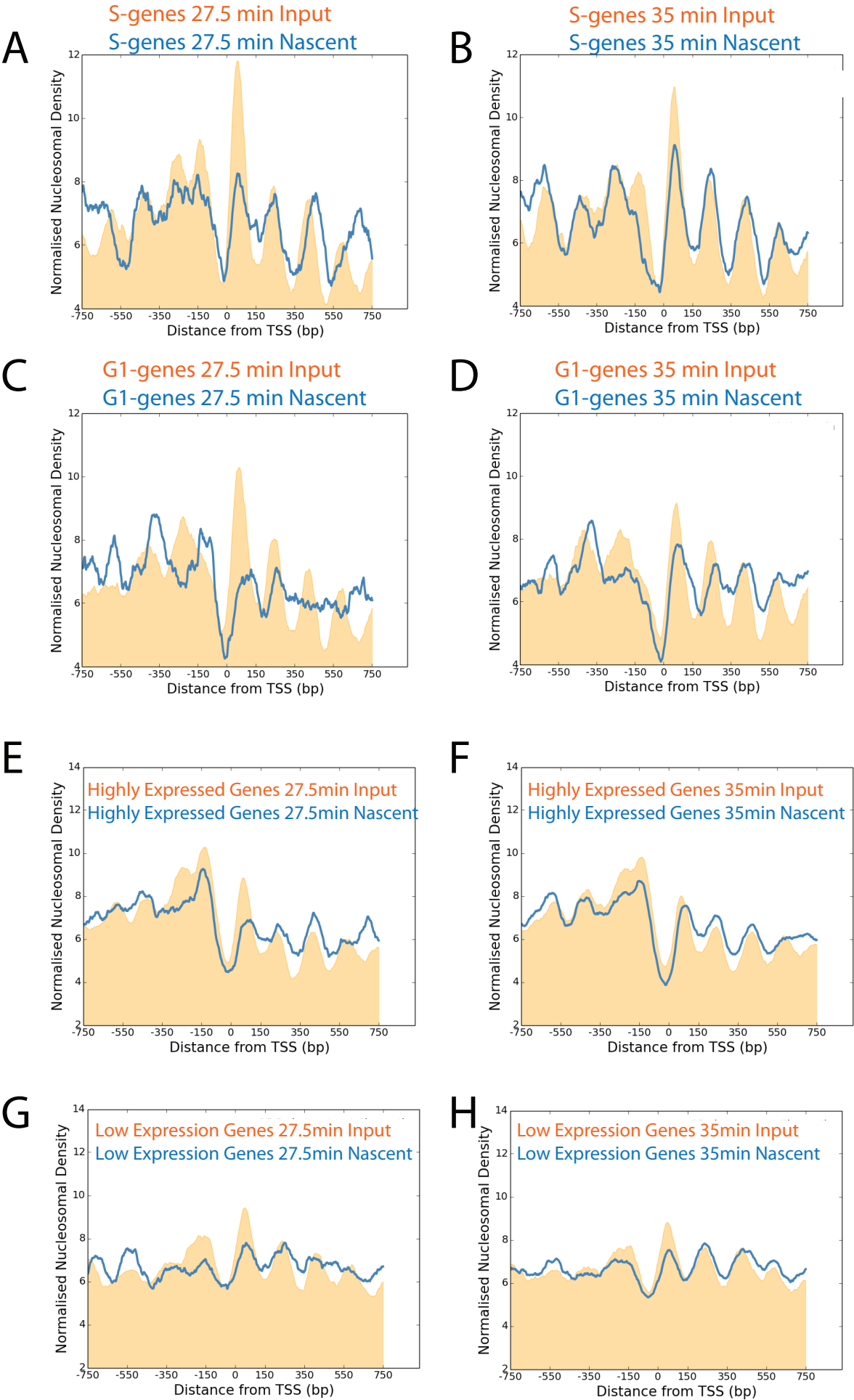

Supplement: SUPPLEMENTARY DATA [file supp_gkw331_nar-00147-m-2016-File.zip › nar-00147-m-2016-File011.pdf]

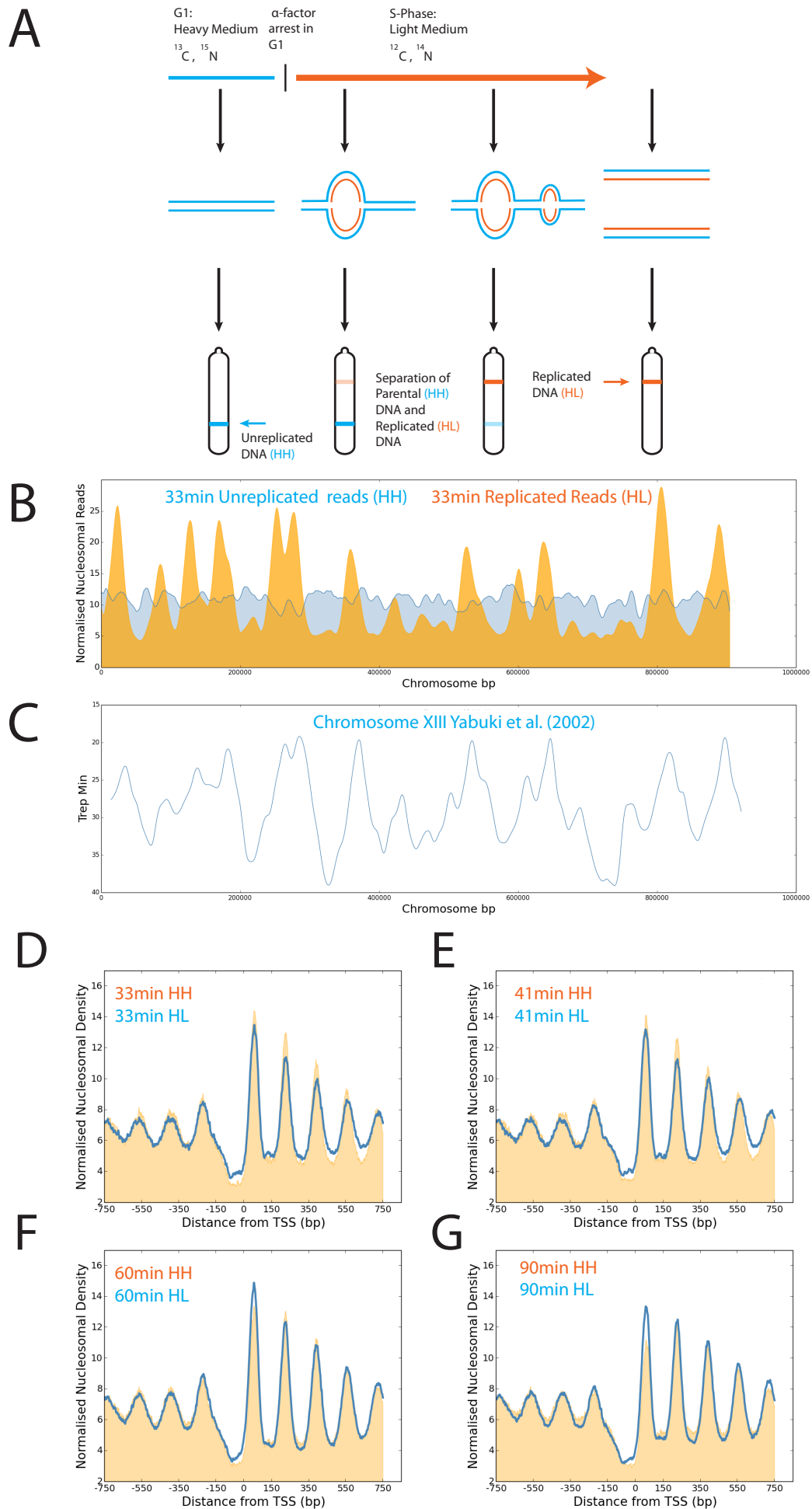

Supplement: SUPPLEMENTARY DATA [file supp_gkw331_nar-00147-m-2016-File.zip › nar-00147-m-2016-File012.pdf]

A

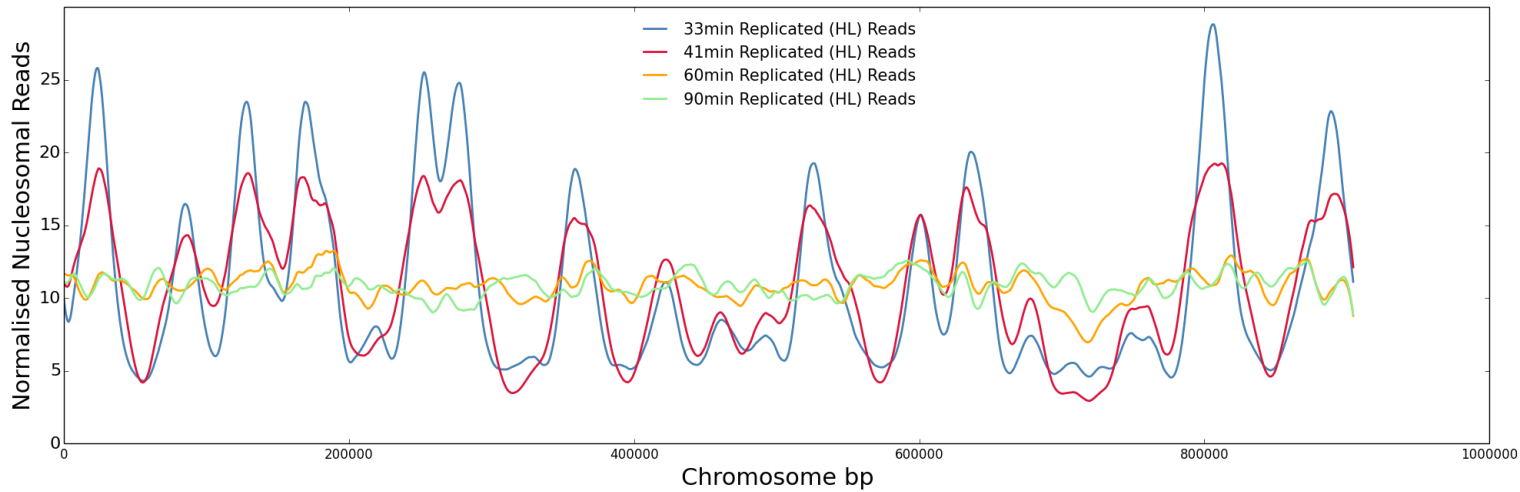

B

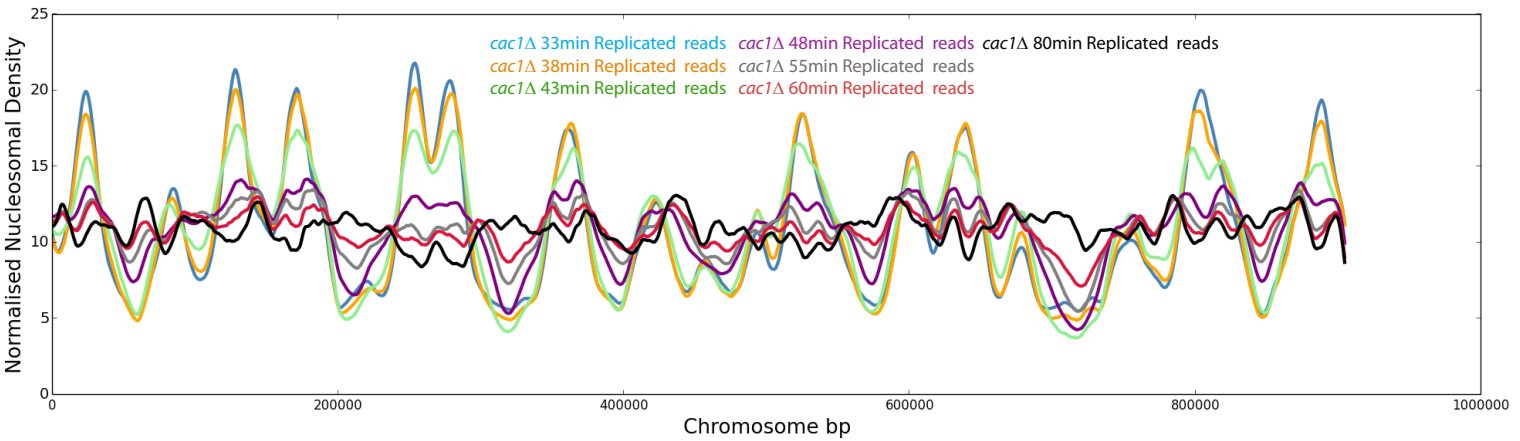

Supplement: SUPPLEMENTARY DATA [file supp_gkw331_nar-00147-m-2016-File.zip › nar-00147-m-2016-File013.pdf]

**A**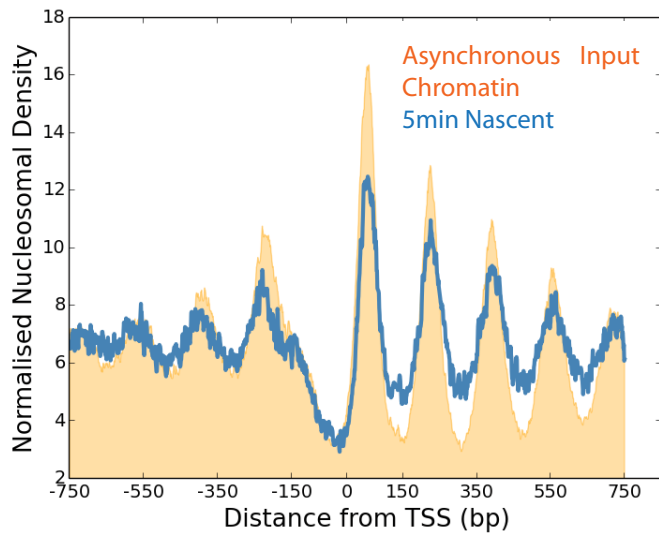**B**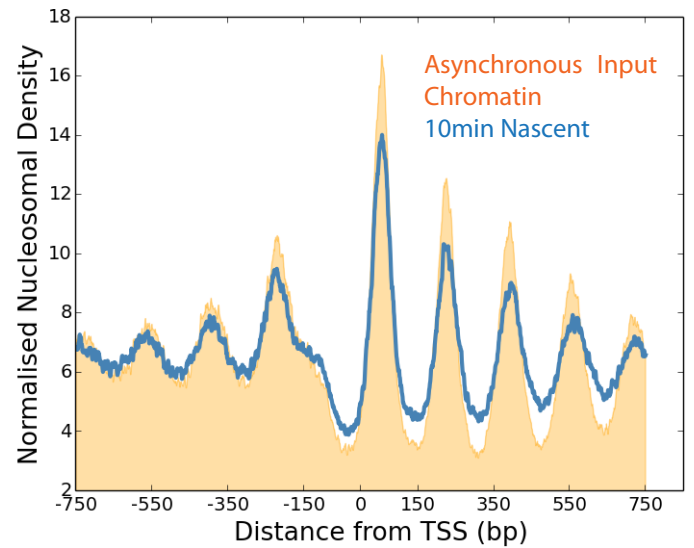**C**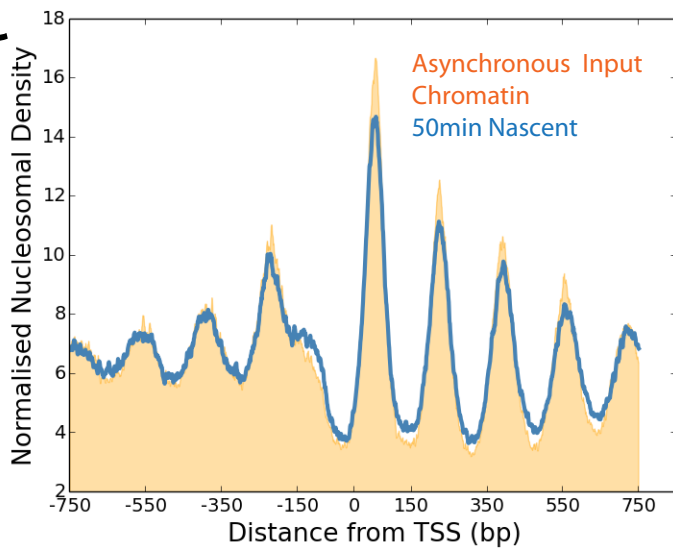

Supplement: SUPPLEMENTARY DATA [file supp_gkw331_nar-00147-m-2016-File.zip › nar-00147-m-2016-File014.pdf]

A

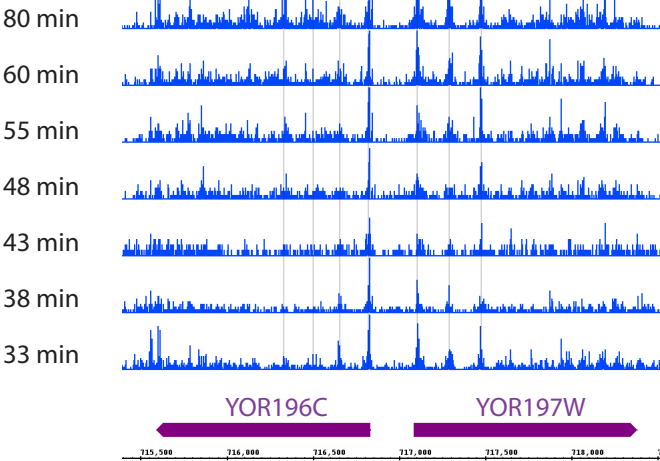

B

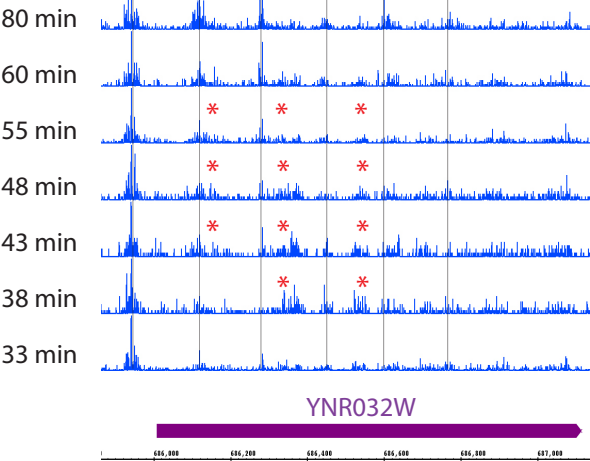

C

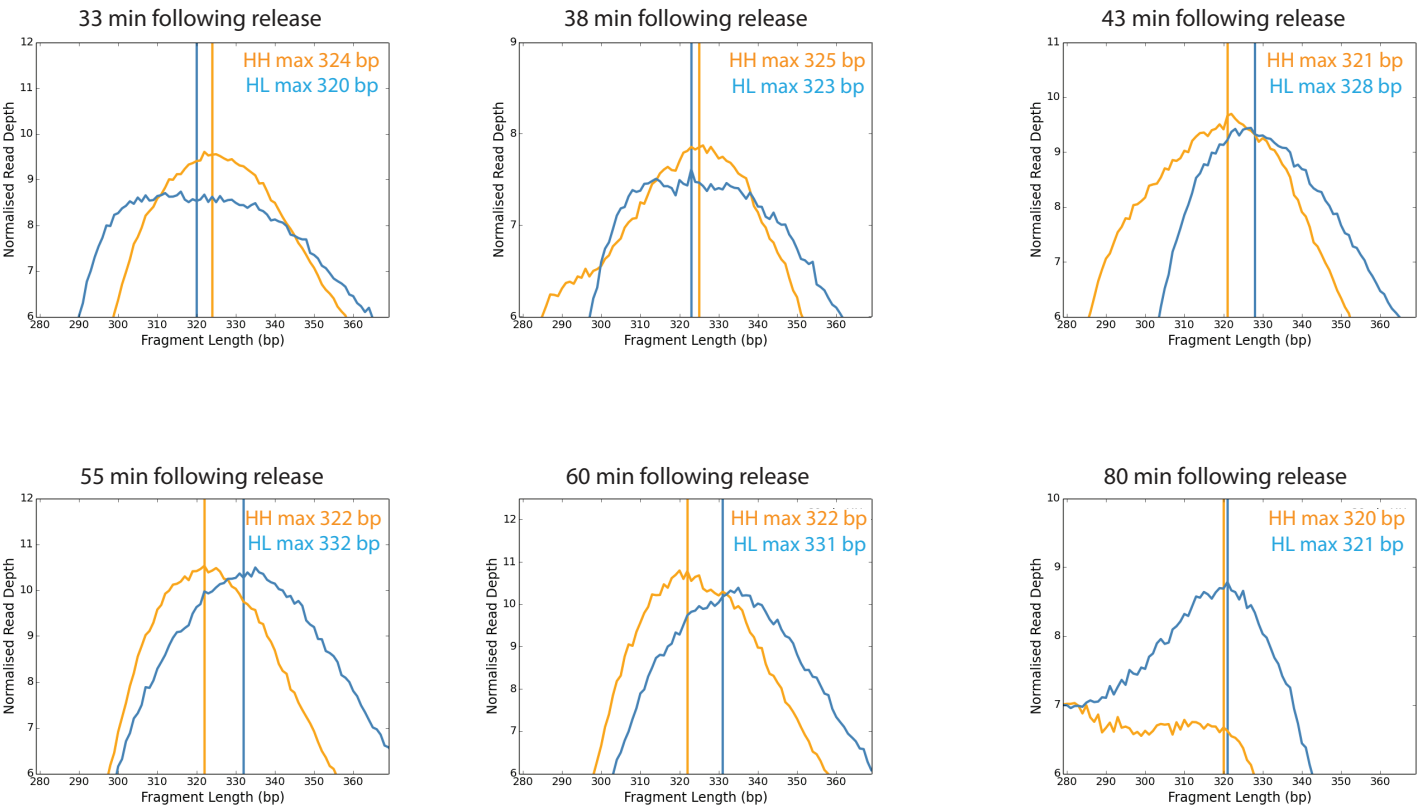

Supplement: SUPPLEMENTARY DATA [file supp_gkw331_nar-00147-m-2016-File.zip › nar-00147-m-2016-File015.pdf]
